# iTRAQ and virus-induced gene silencing revealed three proteins involved in cold response in bread wheat

**DOI:** 10.1038/s41598-017-08069-9

**Published:** 2017-08-08

**Authors:** Ning Zhang, Lingran Zhang, Lei Zhao, Yan Ren, Dangqun Cui, Jianhui Chen, Yongyan Wang, Pengbo Yu, Feng Chen

**Affiliations:** Agronomy College/National Key Laboratory of Wheat and Maize Crop Science/Collaborative Innovation Center of Henan Grain Crops, Henan Agricultural University, Zhengzhou, 450002 China

## Abstract

By comparing the differentially accumulated proteins from the derivatives (UC 1110 × PI 610750) in the F_10_ recombinant inbred line population which differed in cold-tolerance, altogether 223 proteins with significantly altered abundance were identified. The comparison of 10 cold-sensitive descendant lines with 10 cold-tolerant descendant lines identified 140 proteins that showed decreased protein abundance, such as the components of the photosynthesis apparatus and cell-wall metabolism. The identified proteins were classified into the following main groups: protein metabolism, stress/defense, carbohydrate metabolism, lipid metabolism, sulfur metabolism, nitrogen metabolism, RNA metabolism, energy production, cell-wall metabolism, membrane and transportation, and signal transduction. Results of quantitative real-time PCR of 20 differentially accumulated proteins indicated that the transcriptional expression patterns of 10 genes were consistent with their protein expression models. Virus-induced gene silencing of Hsp90, BBI, and REP14 genes indicated that virus-silenced plants subjected to cold stress had more severe drooping and wilting, an increased rate of relative electrolyte leakage, and reduced relative water content compared to viral control plants. Furthermore, ultrastructural changes of virus-silenced plants were destroyed more severely than those of viral control plants. These results indicate that Hsp90, BBI, and REP14 potentially play vital roles in conferring cold tolerance in bread wheat.

## Introduction

Cold stress is one of the major abiotic stresses, as it adversely affects the growth and development of plants and significantly constrains the spatial distribution of plants and agricultural productivity^1^. Cold stress prevents the expression of the full genetic potential of plants via direct inhibition of metabolic reactions and indirect cold-induced osmotic (chilling-induced inhibition of water uptake and freezing-induced cellular dehydration), and oxidative stress^1^. Plants adopt several strategies to cope with this adverse condition, such as raising the level of chaperones and antioxidants, producing more energy by activation of primary metabolisms, and maintaining osmotic balance by altering membrane structure^2–4^. Many overwintering plants, including important crop species such as wheat, rye, and barley, are capable of adapting to low (but not freezing) temperatures (LT) via precise reprogramming of gene expression, e.g., transcription factors, chaperones, metabolic enzymes, late embryogenesis-abundant (LEA) proteins, dehydrins, and antioxidative enzymes^5, 6^. This process of acquiring freezing tolerance is known as cold acclimation (CA)^7, 8^. Overwintering plants acquire freezing tolerance and are capable of surviving under persistent freezing conditions^9^. Acclimation to cold stress is mediated via intense changes in gene expression that translate into alterations in the compositions of the transcriptome, proteome, and metabolome^1, 6, 10^. Due to the regulation of gene expression at transcriptional, post-transcriptional, translational, and post-translational levels^11, 12^, the expression profiles of accumulated proteins are often poorly correlated with their corresponding mRNAs, e.g., in rice^13^, *Arabidopsis* ^9^, and wheat^14^. Considering the facts that proteins are the direct agents of plant stress response^15^, the investigation of dynamic changes in plant proteomes is of great importance.

In recent years, the conventional 2-dimensional electrophoresis (2-DE) and 2-dimensional differential gel electrophoresis (2D-DIGE) followed by mass spectrometry (MS) have been widely used to identify proteome alterations related to chilling and freezing stress in different kinds of plants including barley, soybean, *Arabidopsis thaliana*, rice, wheat, and tobacco^9, 13, 16–21^. However, traditional 2-DE approaches show low identification rates for proteins, inaccurate quantification of different proteins, poor reproducibility, and the difficulty in separating hydrophobic restive factors^22^. Isobaric tagging for relative and absolute quantitation (iTRAQ) is a recently developed and powerful technique that can identify a higher number of proteins and provide more reliable quantitative information^23^. This method has been used to identify abiotic stress responsive-proteins in wheat. Via iTRAQ, the first shotgun wheat proteomics study was conducted to investigate the protein responses to drought^24^. A new metabolic pathway in wheat seedling growth under hydrogen peroxide stress was revealed via iTRAQ-based quantitative proteomic analysis^25^. Proteomic analysis was performed using iTRAQ to study the drought adaptability of roots in 2 wheat cultivars with ABA differential response^26^. Hg-responsive proteins were identified in wheat seedlings using iTRAQ analysis^27^. To our knowledge, no proteomic iTRAQ analysis of the response of common wheat to cold stress has been reported.

Winter wheat is one of the most important crops in most wheat regions of the world. In these areas, frost injury during winter could be particularly destructive. However, limited success has been achieved in developing freezing tolerant cultivars by wheat breeders across the world. Besides the unpredictable nature of frost injury under field conditions, this difficulty may be partly due to the participation of different genes in cold tolerance. Understanding the mechanisms of wheat response to cold stress is crucial for breeding new frost-resistant cultivars and decreasing the risk of crop failure in cold areas^10^. Thus, it is important to identify cold-responsive proteins with potential uses in crop improvement and breeding^28^. In our previous study, a recombinant inbred line (RIL) winter wheat population was utilized to reveal cold-responsive proteins based on 2-DE, and a total of 20 cold-responsive protein candidates were successfully identified. Furthermore, the PAP6-like protein gene was functionally verified to be related to cold response^29^. However, cold response is controlled by polygenes and phenotypic separation of cold response in this RIL population (not 3:1) suggests that there are multiple genes controlling cold response. Therefore, iTRAQ, which possesses higher flux and wider detection range than 2-DE, was performed to uncover additional cold-responsive proteins in this RIL wheat population. In total, 223 proteins were identified with significantly altered abundance. Furthermore, preliminary functional analysis based on virus-induced gene silencing (VIGS) was conducted on 3 new candidate cold-responsive proteins. A better understanding of the cold-tolerance mechanisms in bread wheat via proteomic discovery of these candidate proteins could produce a wide range of benefits in wheat breeding program.

## Materials and Methods

### Plant materials

**Figure 1:**
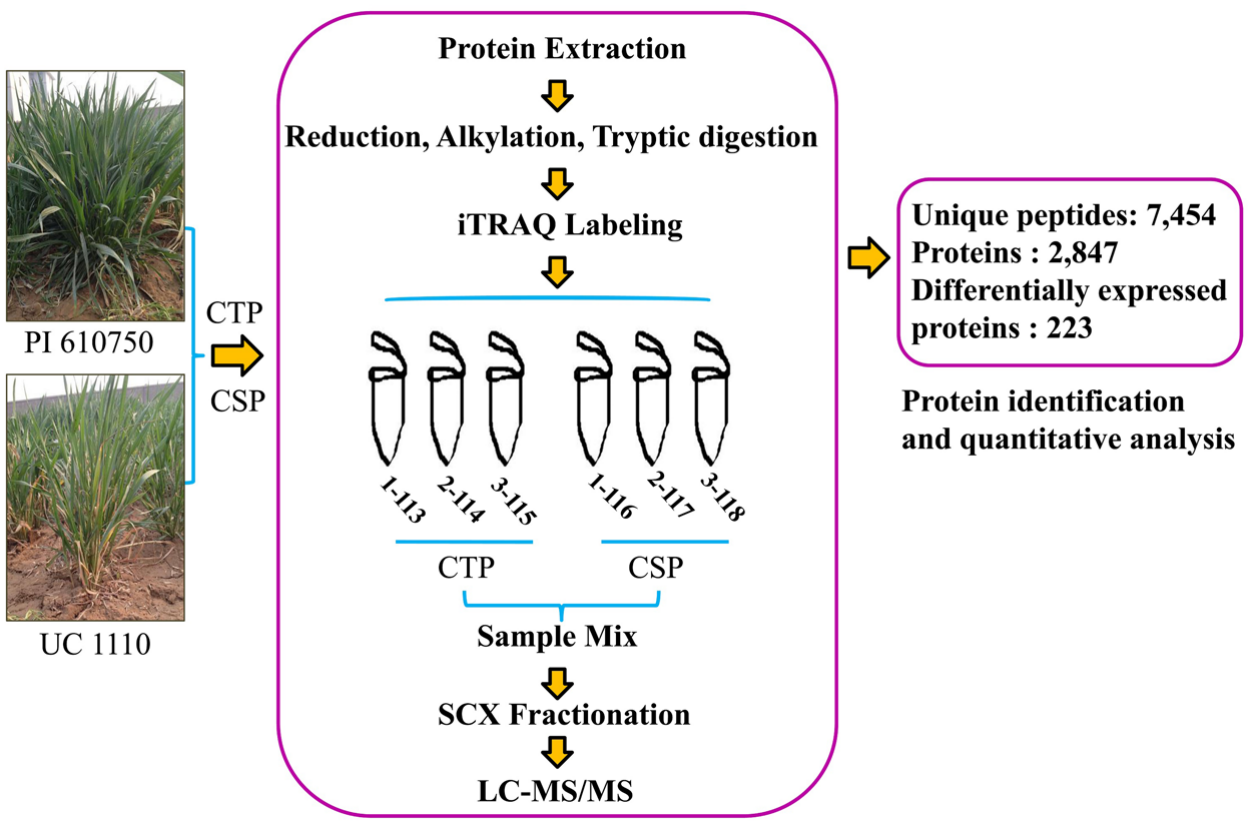
Schematic diagram for identification of cold-responsive proteins via the iTRAQ method.

An overview of the experimental procedures utilized in this study for quantitative proteome analysis is shown in Fig. 1. An F_10_ RIL population (derived from a cross of UC 1110 × PI 610750) encompassing 187 lines was kindly provided by Prof. Jorge Dubcovsky from the University of California, Davis. This cross was planted on October 15, 2013 and October 16, 2014 in Anyang (N 36.1°, E 114.5°), and on October 6, 2013 and October 8, 2014 in Zhengzhou (N 34.9°, E 113.6°). The average monthly growing degree days (GDD) and the investigation of frost injury are shown in Zhang *et al*.^29^. The extent of damage was divided into 4 ranks (3, 2, 1, and 0) from high to low based on phenotypic changes according to the standards of the Wheat Cultivar Approval Committee of the Yellow and Huang wheat region (i.e. sensitive, moderate sensitive, moderate tolerant, cold tolerant, respectively). The wheat parental cultivars UC 1110 (Rank 3) and PI 610750 (Rank 0) differed in cold-tolerance, therefore, the descendants displayed segregation of cold tolerance. Leaves were collected after the investigation of phenotype in Zhengzhou (March 13 of 2015), including one cold-sensitive pool (CSP), and one cold-tolerant pool (CTP). CSP or CTP was composed of an equivalent mixture of leaves from 10 lines of the RIL population with level 3 or 0 under the four environments. Sampled leaves were rapidly frozen in liquid nitrogen, and stored at 80 °C for protein (0.5 g leaves per pool) and RNA (0.3 g leaves per pool) extractions.

### Protein preparation and iTRAQ labeling

Proteins from leaves of CSP and CTP were extracted using the trichloroacetic acid (TCA)/acetone method^30^. Three biological replications were performed, respectively. Protein digestion was performed according to the FASP procedure previously described^31^, and the resulting peptide mixture was labeled using the 8-plex iTRAQ reagent according to the manufacturer’s instructions (Applied Biosystems). For labeling, each iTRAQ reagent was dissolved in 70 μl of ethanol and added to the respective peptide mixture. A 100-μg peptide mixture of each sample was labeled. The samples (biological replicates) were labeled as (CTP-1)-113, (CTP-2)-114, (CTP-3)-115, (CSP-1)-116, (CSP-2)-117, and (CSP-3)-118, and they were multiplexed and vacuum dried (CTP represents control and CSP represents treatment).

### Peptide fractionation with strong cation exchange (SCX) chromatography

The iTRAQ-labeled peptides were fractionated by SCX chromatography using an AKTA Purifier system (GE Healthcare). The dried peptide mixture was reconstituted and acidified with 2 mL buffer A (10 mM KH_2_PO_4_ in 25% of ACN, pH 3.0) and loaded onto a PolySULFOETHYL4.6 × 100 mm column (5 µm, 200 Å, PolyLCInc, Maryland, USA). The peptides were eluted at a flow rate of 1 mL/min with a gradient of 0%–8% buffer B (500 mM KCl, 10 mM KH_2_PO_4_ in 25% of ACN, pH 3.0) for 22 min, 8–52% buffer B at 22–47 min, 52%–100% buffer B at 47–50 min, and 100% buffer B at 50–58 min. Then, buffer B was reset to 0% after 58 min. The elution was monitored by absorbance at 214 nm, and the fractions were collected every 1 min. The collected fractions were desalted on C18 Cartridges (Empore™ SPE Cartridges C18 (standard density), bed I.D. 7 mm, volume 3 mL, Sigma) and concentrated by vacuum centrifugation.

### Liquid chromatography (LC) - tandem mass spectroscopy (MS/MS) analysis by using Q exactive

Each fraction was injected for nanoLC-MS/MS analysis. The peptide mixture was loaded onto a reverse phase trap column (Thermo Scientific Acclaim PepMap100, 100 μm*2 cm, nanoViper C18) connected to the C18-reversed phase analytical column (Thermo Scientific Easy Column, 10 cm long, 75 μm inner diameter, 3 μm resin) in buffer A (0.1% Formic acid) and separated with a linear gradient of buffer B (84% acetonitrile and 0.1% Formic acid) at a flow rate of 300 nl/min controlled by IntelliFlow technology. LC-MS/MS analysis was performed on a Q Exactive mass spectrometer (Thermo Scientific) that was coupled to Easy nLC (Proxeon Biosystems, now Thermo Fisher Scientific). The mass spectrometer was operated in positive ion mode. MS data was acquired using a data-dependent top10 method dynamically choosing the most abundant precursor ions from the survey scan (300–1800 m/z) for HCD fragmentation. Determination of the target value is based on predictive Automatic Gain Control (pAGC). Dynamic exclusion duration was 60 s. Survey scans were acquired at a resolution of 70,000 at m/z 200 and resolution for HCD spectra was set to 17,500 at m/z 200. Normalized collision energy was 30 eV and the underfill ratio, which specifies the minimum percentage of the target value likely to be reached at maximum fill time, was defined as 0.1%. The instrument was run with peptide recognition mode enabled. The mass spectrometry proteomics data have been deposited to the ProteomeXchange Consortium via the PRIDE^32^ partner repository with the dataset identifier PXD004427.

### Protein identification and quantitative analysis

MS/MS spectra were searched using MASCOT engine (Matrix Science, London, UK; version 2.2) embedded into Proteome Discoverer 1.4 software (Thermo Electron, San Jose, CA) run against the UniProt Poacese database (released at September 17, 2015, 1248700 sequences). For protein identification, the following options were used: peptide mass tolerance =  ± 20 ppm; fragment mass tolerance = 0.1 Da; enzyme = trypsin; max missed cleavage = 2; fixed modification: carbamidomethyl (C), iTRAQ8plex (K), iTRAQ8plex (N-term); variable modification: oxidation (M), iTRAQ8plex (Y), and database pattern: decoy, peptide false discovery rate (FDR) ≤ 0.01. For protein quantification, the protein ratios are calculated as the median of only unique peptides of the protein. For the experimental bias, all peptide ratios are normalized by the median protein ratio. The median protein ratio should be 1 after the normalization.

Differentially accumulated proteins were analyzed for significant downregulation or upregulation. Ratio of the abundance of the proteins identified in CSP to that of CTP was used to assess their fold changes. Moreover, one sample t-test was used to identify significant (*p* < 0.05) differences in means between CSP and CTP Differentially accumulated proteins were defined on the basis of thresholds of >1.2- or <0.83-fold change ratios in CSP compared to those of CTP.

### Bioinformatics analysis

In this study, the identified proteins were annotated by searching against Uniprot Poacese database (1248700 sequences, download at September 17, 2015). Then, the differentially accumulated proteins were grouped on the basis of their biological functions using Gene Ontology (GO) terms (http://www.geneontology.org/) and were mapped to the reference authoritative pathways in Kyoto Encyclopedia of Genes and Genomes (KEGG) (http://www.genome.jp/kegg/) to determine the active biological pathways.

### Transcriptional expression analysis by quantitative real-time PCR (qRT-PCR)

Based on the functional category and differential expression fold, twenty of the differentially-abundant proteins were chosen by qRT-PCR. The specific primers were designed using Primer 3.0 (Additional Table S1). Total RNA from CSP and CTP was extracted using the total RNA kit (TaKaRa, Dalian, China). Two-Step PrimeScript^TM^ RT Reagent Kit with gDNA Eraser (Perfect Real Time; TaKaRa) was used for the RT reactions. qRT-PCR was conducted using a Bio-Rad IQ5 Real-Time PCR Detection System. The details of reaction system were shown in Zhang *et al*.^29^. All reactions were performed in triplicates for each sample. The *β*-actin gene (GenBank accession no. AB181991) served as the endogenous control.

### System of virus-induced gene silencing (VIGS)

The wheat cultivar Zhengmai 9023 was used for the VIGS experiment. Primers (Additional Table S1) were designed using software Primer 3.0 software. We generated 188-bp, 198-bp, and 189-bp fragments for Heat shock protein 90 (have 99% identities with common wheat Hsp90.2) (Hsp90) (F2CU34), Bowman-Birk type protease inhibitor (BBI) (M7YVE8) and REP14 (have 95% identities with wheat Wcor15) (Q8S385), respectively. Vector constructs were performed as previously described^33^. Plasmids linearization^34^, as well *in vitro* transcripts and mix^35^ according to the method of Zhang *et al*.^29^. The original BSMV: BSMV_0_ was constructed from *α*, *β*, and *γ* RNA derived from the original empty pSL038-1 vector, and acted as the viral control. BSMV: PDS (GenBank: FJ517553.1), mentioned by Zhou *et al*. (2011)^36^, was used in our study to monitor the time course of VIGS (positive control), which was shown in Zhang *et al*.^29^. A volume corresponding to 3 g viral RNA was rub inoculated onto the second leaf of silenced seedlings at the 2–3 leaf stage^36^. The third and fourth leaf tissues (0.3 g) were collected from each treatment group at 14 days post-inoculation (dpi) for qRT-PCR to determine the efficiency of silencing of Hsp90, BBI, and REP14, respectively. Besides, the successful rates of the plants (20 seedlings per biological replicate) inoculated with different BSMVs were recorded at 14 dpi, and three independent biological replicates were performed for each BSMV.

### Imposition of freezing stress and assessment of physiological parameters

The seeds of wheat cultivar Zhengmai 9023 were immersed and sterilized with 1% (w/v) H_2_O_2_ for 0.5 h and then were thoroughly washed with distilled water. The sterilized seeds were covered with water in petri dishes for 24 h to germinate. The uniform seedlings (plant height ≈ 3.5 ± 0.1 cm) were transferred into plastic pots holding 800 g of potting mixture. Each pot contained 6 plants and all of the seedlings were maintained in a growth chamber at 23 °C under 16/8 h light/dark photoperiod with 5500 Lx light intensity and relative humidity of 70%. Each plant was observed as an independent biological replicate, and totals of 10 biological replicates were investigated for each treatment. For each experiment, two subsets of plants were provided. The control set of plants was maintained at normal conditions and freezing stress was imposed on the other set of plants at −5 °C for 5 days.

In order to assess effects of freezing stress, the rate of relative electrolyte leakage^13^ and the leaf relative water content (RWC)^37^ were estimated. After freezing stress, stress responses were assessed by taking leaf samples from the uppermost fully expanded leaves of both stressed and non-stressed plants (non-stressed and non-silenced plants, freeze-stressed and non-silenced plants, freeze-stressed BSMV_0_-treated plants, and freeze-stressed BSMV_Hsp90_, BSMV_BBI_, and BSMV_REP14_-inoculated plants). Three independent biological replicates were performed for each measurement.

### Transmission electron microscopy

After exposure to cold stress for 5 days, we observed the ultrastructure changes caused by virus infection. Transmission electron microscopy (TEM) was used to evaluate the structure of non-stressed and non-silenced plants, freeze-stressed and non-silenced plants, freeze-stressed BSMV_0_-treated plants, and freeze-stressed BSMV_BBI_-inoculated plants (as one example). Sample sections from the middle portion of the third developed leaf (1 mm^2^ of top middle section of the fully expanded leaf) were excised, fixed in cold 4% (v/v) glutaraldehyde in 0.1 M potassium-phosphate buffer (PBS; pH 7.2), vacuum-infiltrated until the material sank, and left overnight at 4 °C. The samples were then dehydrated in a graded alcohol series and embedded in resin^38^. Sample semi-micro sections of 0.2-*μ*m thickness were generated using an LKB11800 Pyramitome (Sweden). These sections were then examined using a transmission electron microscope (model 7500; Hitachi, Tokyo, Japan) at 80 kV. At least 3 sections from each treatment were examined.

### Statistical analysis

All experiments in this study were repeated independently in triplicate. A one-way analysis of variance (ANOVA) using the SPSS 17.0 statistical software and Duncan’s multiple range test (DMRT) was used to identify significant (*p* < 0.05) differences between group means.

## Results

### Identification and classification of differentially accumulated proteins of CSP compared with CTP

**Figure 2:**
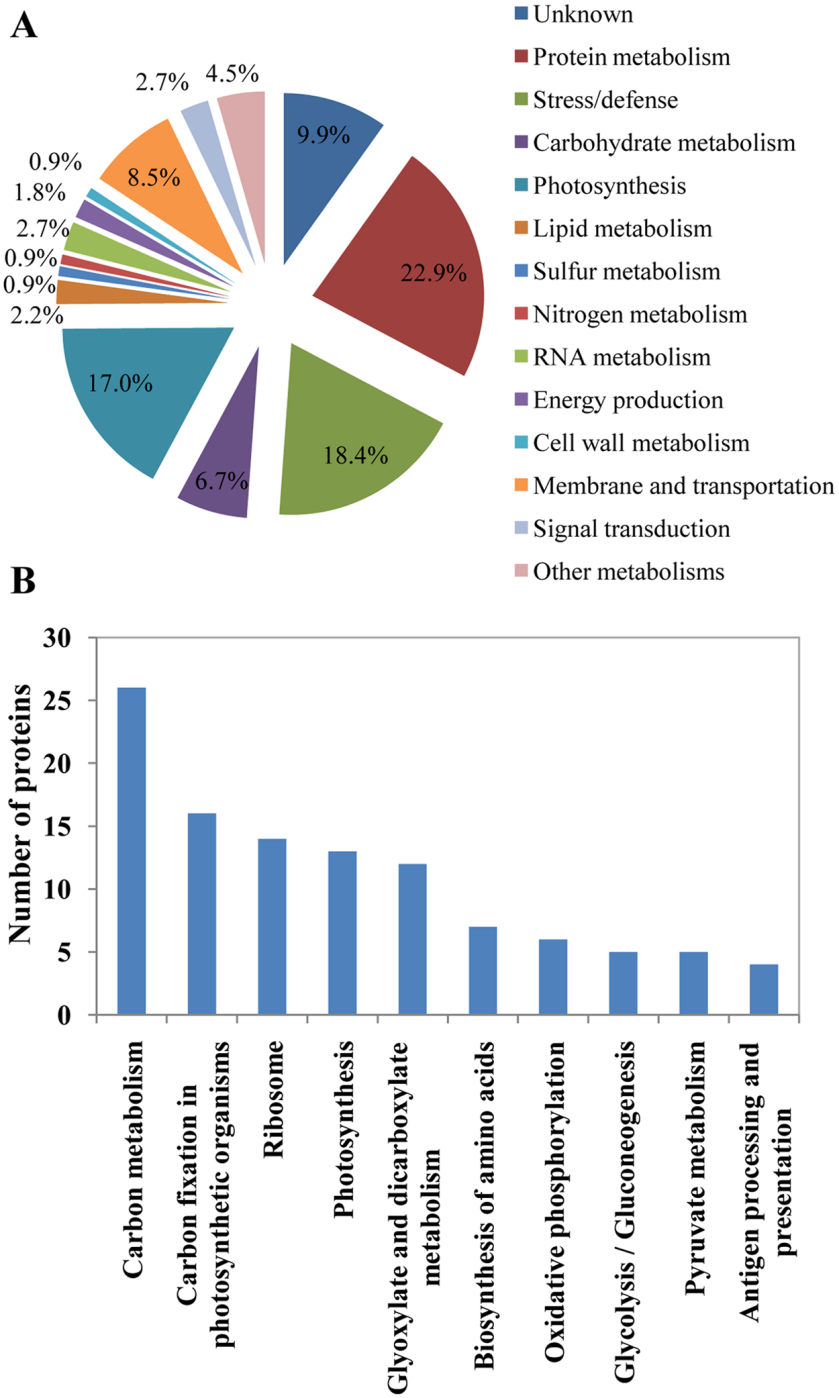
Classification (**A**) of the identified differentially accumulated proteins and the number distribution (**B**) of the identified proteins involved in signaling pathways by KEGG.

**Figure 3:**
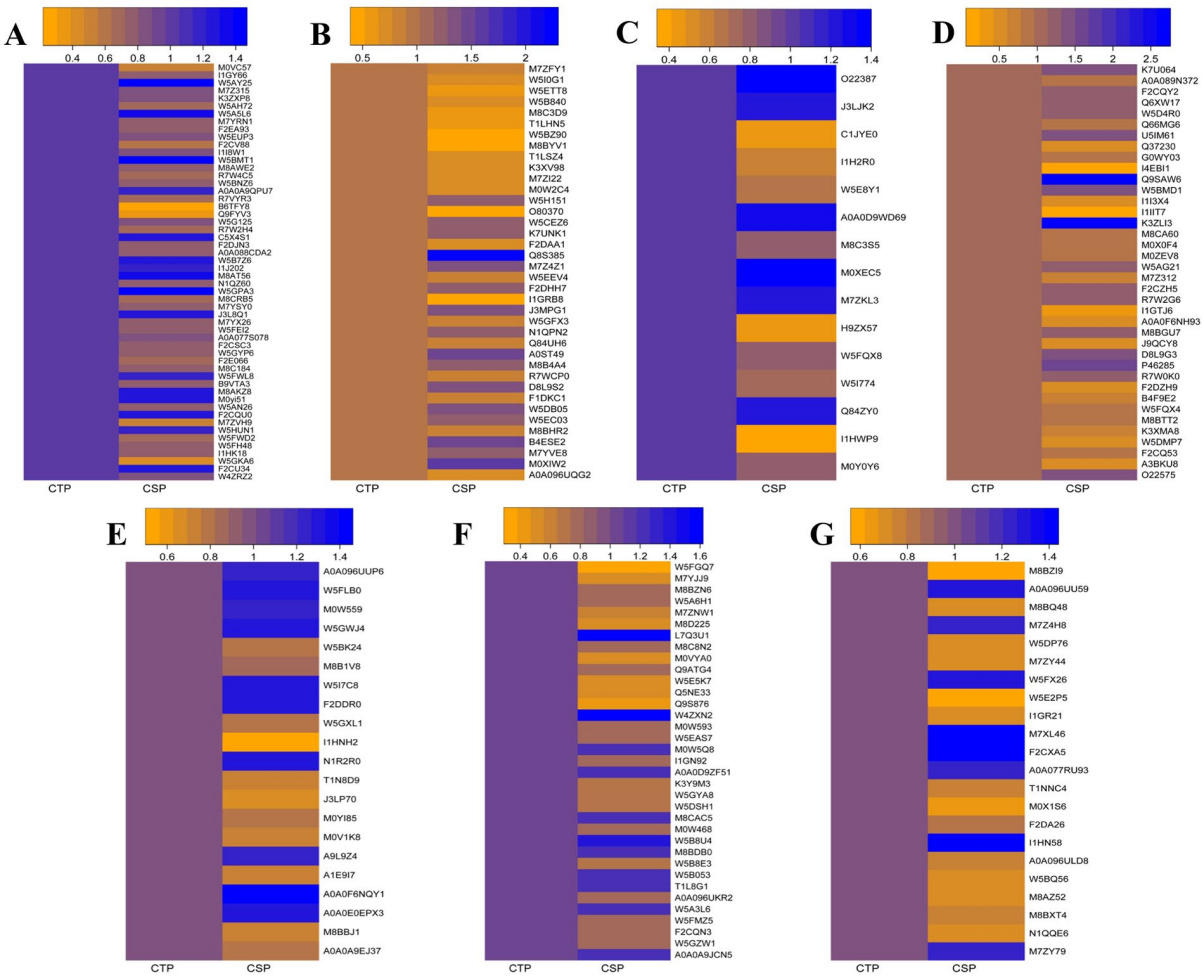
Abundance of the identified differentially accumulated proteins between CSP and CTP. The differentially accumulated proteins were involved in protein metabolism (**A**), stress/defense (**B**), carbohydrate metabolism (**C**), photosynthesis (**D**), lipid metabolism (**E**), sulfur metabolism (**E**), nitrogen metabolism (**E**), RNA metabolism (**E**), energy production (**E**), cell-wall metabolism (**E**), membrane and transportation (**F**), signal transduction (**F**), others (**F**) and unknown (**G**).

In order to exclude the difference of genetic background between two independent cultivars/lines, two mixed pools CSP and CTP (control) from a RIL population were used to identify proteome profiles via multiplex iTRAQ-based quantitative proteomic and LC-MS/MS methods. The phenotypes of lines composed CTP and CSP are shown in Zhang *et al*.^29^. A total of 11,006 peptides, 7,454 unique peptides, and 2,847 proteins (unique peptides ≥ 1) were identified (Fig. 1; Additional files 1 and 2). Altogether, 223 differentially accumulated proteins were identified with Ratio > 1.2-fold or < 0.83-fold (Additional file 3). The functions of the differentially accumulated proteins were categorized into several main groups based on their GO annotations (Additional file 4), including protein metabolism (51, 22.9%), stress/defense (41, 18.4%), photosynthesis (38, 17.0%), carbohydrate metabolism (15, 6.7%), lipid metabolism (5, 2.2%), sulfur metabolism (2, 0.90%), nitrogen metabolism (2, 0.90%), RNA metabolism (6, 2.7%), energy production (4, 1.8%), cell wall metabolism (2, 0.9%), membrane and transportation (19, 8.5%), signal transduction (6, 2.7%), other metabolic processes (10, 4.5%) and unknown biological processes (22, 9.9%) (Additional Table S2 and Fig. 2A). According to KEGG analysis, the proteins with significant changes are mainly involved in carbon metabolism, carbon fixation in photosynthetic organisms, and ribosome (top 3) pathways (Additional file 5 and Fig. 2B). Furthermore, 140 out of the 223 differentially accumulated proteins showed decreased protein abundance in CSP when compared with CTP. This decreased protein abundance was especially evident in the proteins involved in protein metabolism, photosynthesis, RNA metabolism, cell membrane, and transportation (Additional Table S2 and Fig. 3). Previously, 23 differentially accumulated proteins between the CSP-1 and CTP-1 were identified via 2-DE. These proteins represented 6 functional categories^29^ (Additional Table S3). Using the same materials, a total of 223 differentially accumulated proteins were identified via iTRAQ in this study. These were involved in 12 functional groups (Additional Table S3); for instance, proteins associated with lipid metabolism, sulfur metabolism, RNA metabolism, cell wall metabolism, membrane and transportation, and signal transduction were also identified. Additionally, more than 50% of the differentially accumulated proteins identified by 2-DE was also recognized by iTRAQ.

### Comparison of expression patterns of identified proteins at the mRNA and protein levels

**Figure 4:**
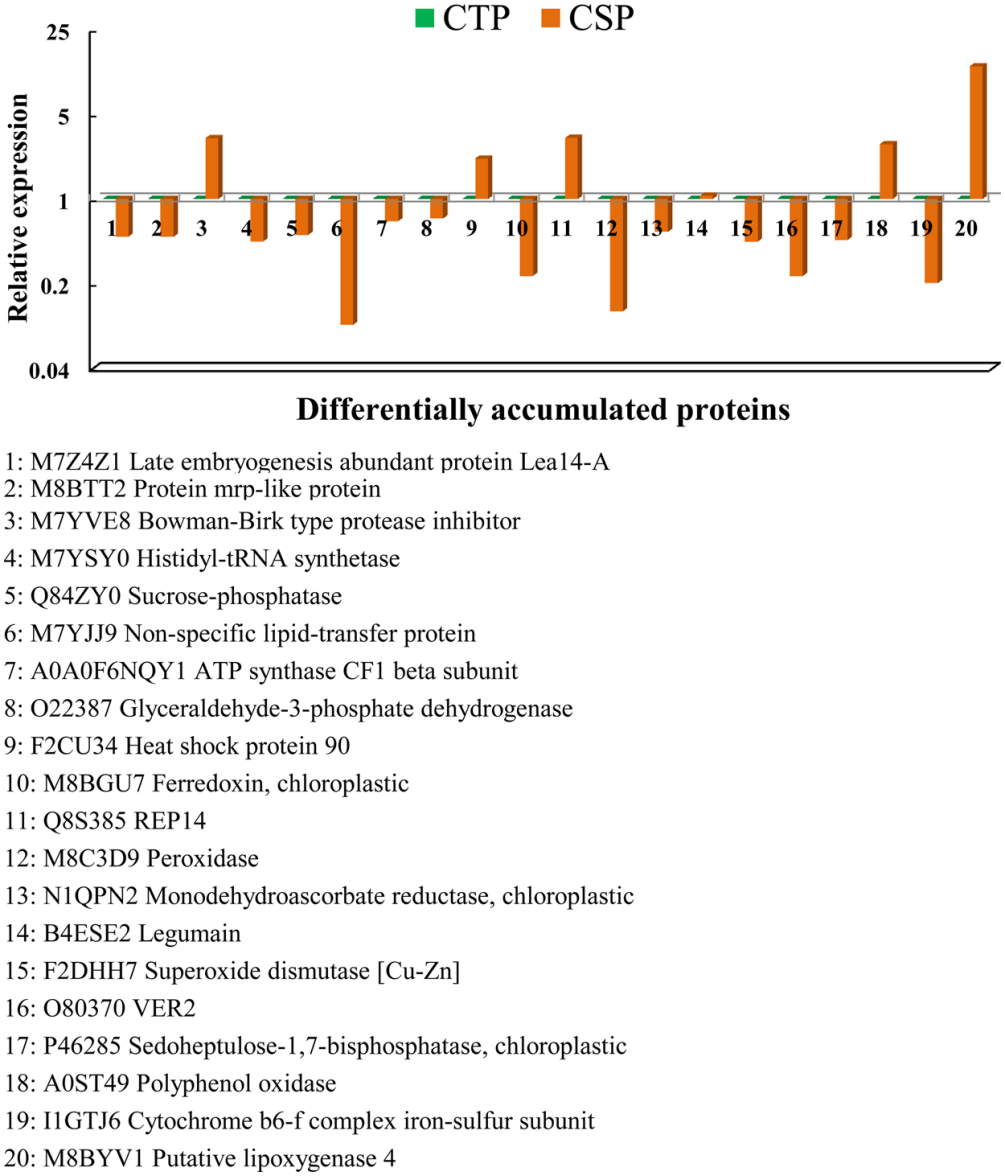
Expression profiles of the 20 cold-responsive candidate genes via qRT-PCR. Note: CSP compared with CTP.

Seven of the downregulated proteins [i.e., protein mrp-like protein (M8BTT2), histidyl-tRNA synthetase (M7YSY0), non-specific lipid-transfer protein (M7YJJ9), peroxidase 12(M8C3D9), VER2 (O80370), cytochrome b6-f complex iron-sulfur subunit, chloroplastic-like (I1GTJ6), putative lipoxygenase 4 (M8BYV1)] and thirteen of the upregulated proteins [i.e., Late embryogenesis abundant protein Lea14-A (M7Z4Z1), Bowman-Birk type protease inhibitor (M7YVE8), plastid 3-phosphoglycerate kinase, partial (Q84ZY0), ATP synthase CF1 beta subunit (A0A0F6NQY1), glyceraldehyde-3-phosphate dehydrogenase (O22387), Heat shock protein 90 (F2CU34), ferredoxin, chloroplastic (M8BGU7), Q8S385(REP14), monodehydroascorbate reductase, chloroplastic (N1QPN2), legumain (B4ESE2), superoxide dismutase [Cu-Zn] (F2DHH7), sedoheptulose-1,7-bisphosphatase, chloroplastic (P46285), polyphenol oxidase (A0ST49)] were chosen by qRT-PCR to investigate the expression changes at the RNA level that could be correlated with cold tolerance. The results show that the expression of legumain (B4ESE2) (1.06-fold) exhibited no significant difference between CSP and CTP. A comparison of the expression patterns at the RNA and protein levels indicate that the transcriptional expression patterns of 10 genes were consistent with their protein expression models, whereas the remaining 9 genes displayed poor consistency between the transcriptional and translational levels in CSP when compared with CTP (Fig. 4).

### VIGS of the 3 candidate protein genes as well as phenotypic and physiological changes of silenced plants under freezing stress

**Figure 5:**
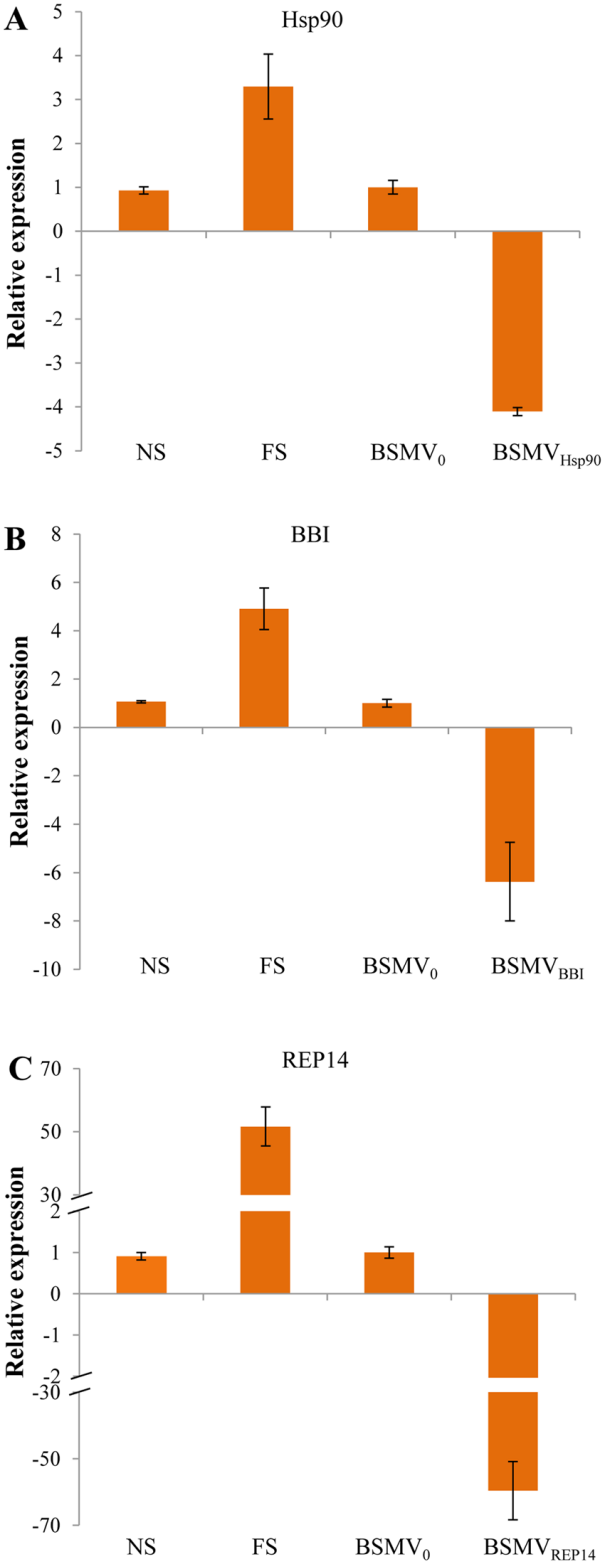
Relative expression of the three candidate genes in differently treated wheat plants via qRT-PCR. Expression of BSMV_Hsp90_, BSMV_BBI_, and BSMV_REP14_ in non-stressed non-silenced (NS), freeze-stressed non-silenced (FS), and silenced plants BSMV_Hsp90_, BSMV_BBI_, and BSMV_REP14_ at 14 dpi were calibrated to the mean levels of expression of the genes in the BSMV_0_-treated plants. Bars represent standard errors of triplicate experiments.

Three novel protein genes (i.e., Hsp90, BBI, and REP14) were identified by iTRAQ that differ from PAP6-like, spots N °6 and N °21 as previously reported in Zhang *et al*.^29^. These genes were selected for further functional analysis via VIGS. Both mRNA and protein expression of the 3 candidate genes increased in this study, and these genes were therefore selected to test whether a single candidate protein gene could enhance cold tolerance in wheat. VIGS was performed to evaluate the roles of Hsp90, BBI, and REP14 in the response of wheat to cold stress conditions. Under VIGS test, the successful rates of the plants inoculated with BSMV_0_, BSMV_Hsp90_, BSMV_BBI_, BSMV_REP14_ and BSMV_PDS_ at 14 dpi were 86.7%, 85%, 83.3%, 85%, and 90%, respectively (Additional Figure S1). qRT-PCR was performed to determine the transcript levels of Hsp90, BBI, and REP14 in silenced plants, the viral controls, non-stressed non-silenced (NS), and freeze-stressed non-silenced (FS) plants at 14 dpi. The transcript levels of the 3 protein genes were all significantly reduced in silenced plants compared to plants inoculated with only BSMV_0_ (Fig. 5). The average Hsp90, BBI, and REP14 transcript levels were reduced 4.1-fold, 6.4-fold, and 59.6-fold in BSMV_Hsp90_, BSMV_BBI_, and BSMV_REP14_-inoculated plants, respectively. The average Hsp90, BBI, and REP14 transcript levels were increased 3.3-fold, 4.9-fold, and 51.6-fold in FS, respectively, when compared to BSMV_0_-treated plants. The transcript levels of Hsp90, BBI, and REP14 in NS (0.93-fold, 1.06-fold, and 0.91-fold) were not significantly different from viral control plants (Fig. 5A–C).

**Figure 6:**
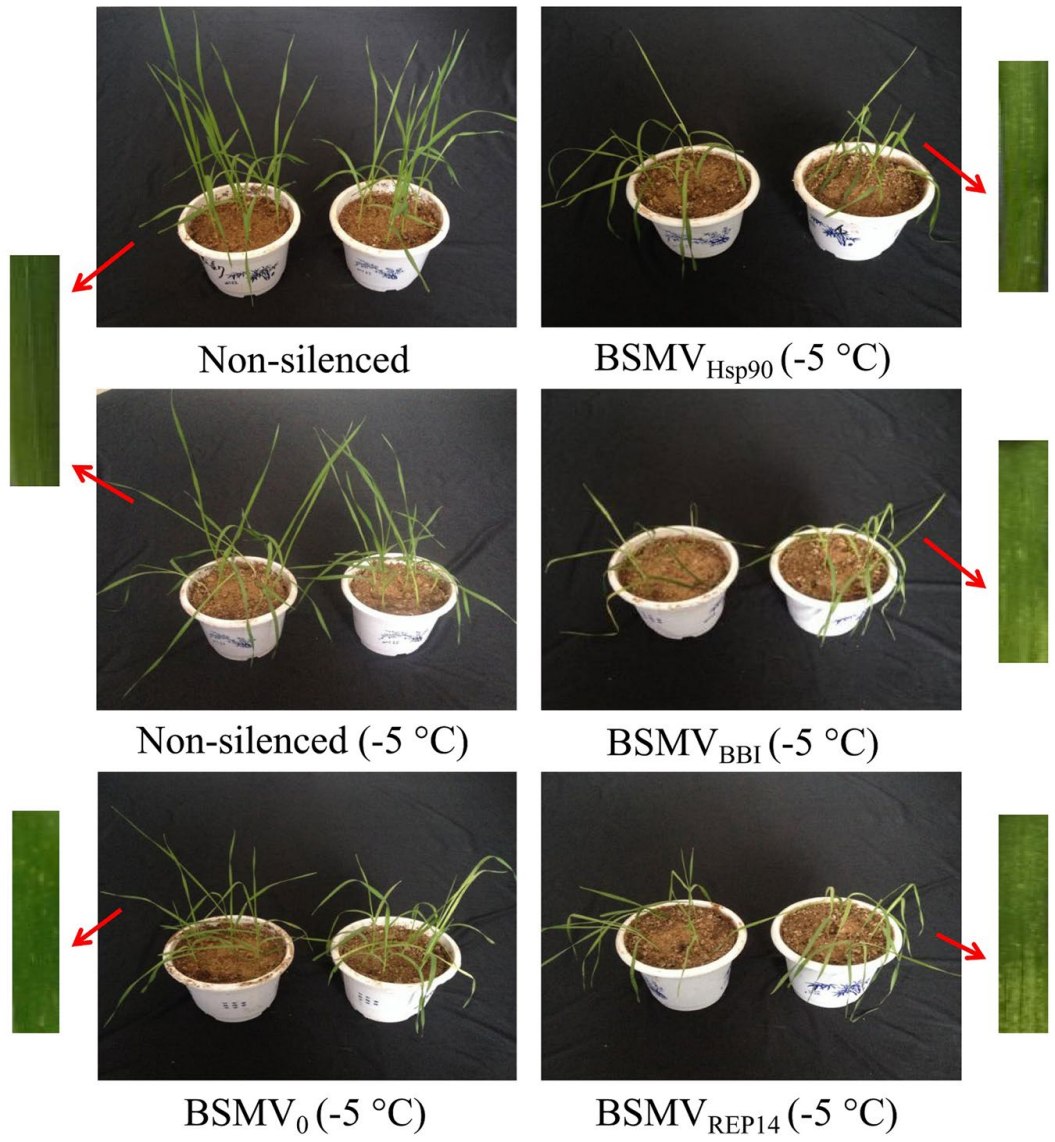
Phenotypes of the virus-infected wheat plants with BSMV RNA transcripts under the freezing stress at day 5. **N**on-silenced plant served as control, BSMV_0_, BSMV_Hsp90_, BSMV_BBI_, and BSMV_REP14_-treated plant compared to the control (leaf phenotypes). Freeze-stressed BSMV_0_-inoculated plants served as control. Non-silenced non-stressed, non-silenced freeze-stressed (−5 °C) plants, and freeze-stressed BSMV_Hsp90_, BSMV_BBI_, and BSMV_REP14_-treated plants were included for comparison of phenotypes. Note: The depressed vigour of plants silenced for Hsp90, BBI, and REP14 were compared to the viral control plants.

Phenotypes of the plants were observed during the entire course of the experiment (from 1 to 19 days). After rub inoculation with BSMV constructs, slight chlorosis was observed in the viral controls and in all of the silenced plants due to the plant’s response to virus infection (Fig. 6). This phenomenon is prevalent in previous reports of VIGS studies on the leaf rust resistance gene Lr21^35^ as well as the *Arabidopsis thaliana* gene homologues Era1, Cyp707a, Sal1^37^, and WRKY53^39^ in wheat. The drooping and wilting symptoms were observed in plants after 5 days of freezing stress (Fig. 6). Leaves of the freeze-stressed BSMV_Hsp90_, BSMV _BBI_, and BSMV_REP14_-treated plants showed a distinctly higher level of drooping and wilting in comparison to plants from the other freeze-stressed treatments.

**Figure 7:**
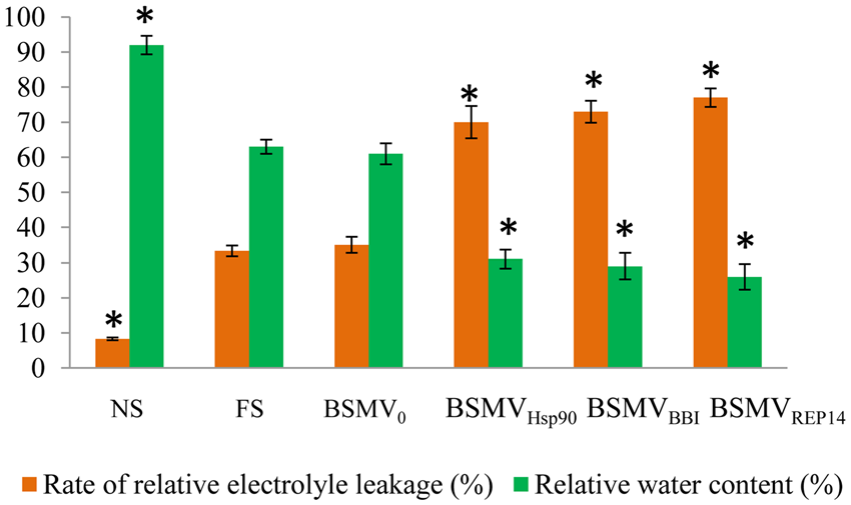
Comparison of the rate of relative electrolyte leakage and the leaf relative water content among freeze-stressed wheat plants. NS, non-stressed non-silenced; FS, freeze-stressed non-silenced; BSMV_0_, freeze-stressed viral control plants; BSMV_Hsp90_, BSMV_BBI_, and BSMV_REP14_, freeze-stressed silenced plants. Values are means (±SE) of three observations. Bars represented standard errors of triplicate experiments. Significant differences between the control and all other plants were determined by performing a one-way analysis of variance (ANOVA). Asterisks denoted significant difference from the viral control plants (*P* < 0.05).

After 5 days of exposure to −5 °C, the rates of relative electrolyte leakage were examined in all treatment groups (Fig. 7). The FS plants exhibited markedly increase in the rates of relative electrolyte leakage relative to the NS plants. The FS plants did not differ remarkably from the stressed viral control, indicating that virus inoculation had no effect on the rates of relative electrolyte leakage in the plants. Additionally, plants silenced for Hsp90, BBI, and REP14 also showed a significant increase in the rates of relative electrolyte leakage as compared to FS and viral control plants. Furthermore, the impact of silencing on plant water status under cold limitation was examined (Fig. 7). Freeze-stressed BSMV_0_-treated plants and FS plants did not have significant differences in RWC, whereas the FS plants exhibited drastically reduce in RWC when compared to the NS plants. Similarly, in comparison to freeze-stressed BSMV_0_-treated plants, the freeze-stressed BSMV_Hsp90_, BSMV_BBI_, and BSMV_REP14_-treated plants had a significant reduction in RWC.

### Ultrastructural changes in leaves of wheat with silenced BBI gene under freezing stress

**Figure 8:**
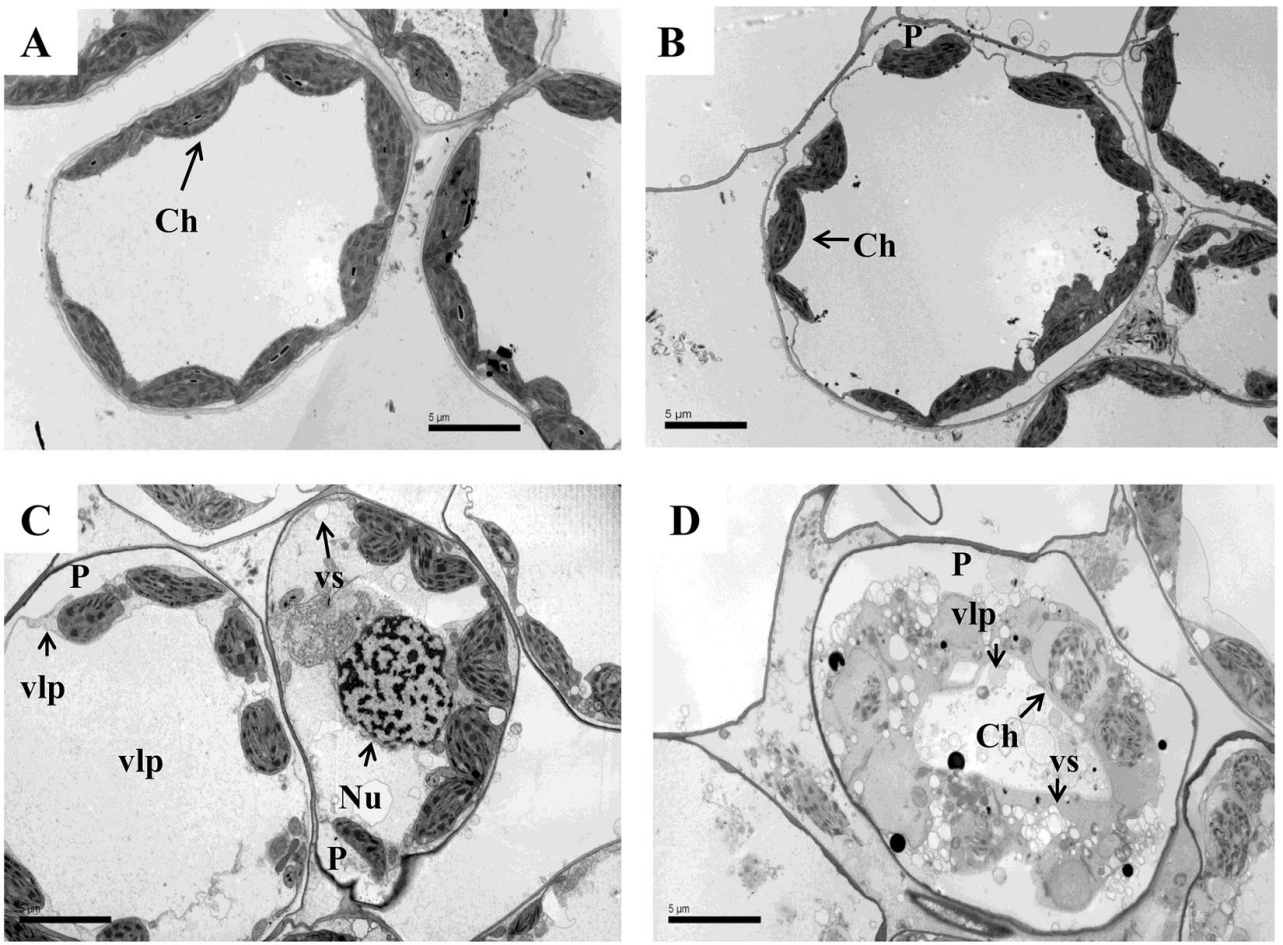
Transmission electron micrographs of mesophyll cells among freeze-stressed wheat plants. (**A**) Non-silenced non-stressed; (**B**) non-silenced freeze-stressed; (**C**) freeze-stressed BSMV_0_-treated plants; (**D**) freeze-stressed BSMV_BBI_-treated plants. Labels: Ch, chloroplast; Gr, granum; Nu, nucleus; P, plasmolysis; SG, starch grain; vlp, virus-like particles; and vs, small vesicle. Bars: 5 *μ*m.

**Figure 9:**
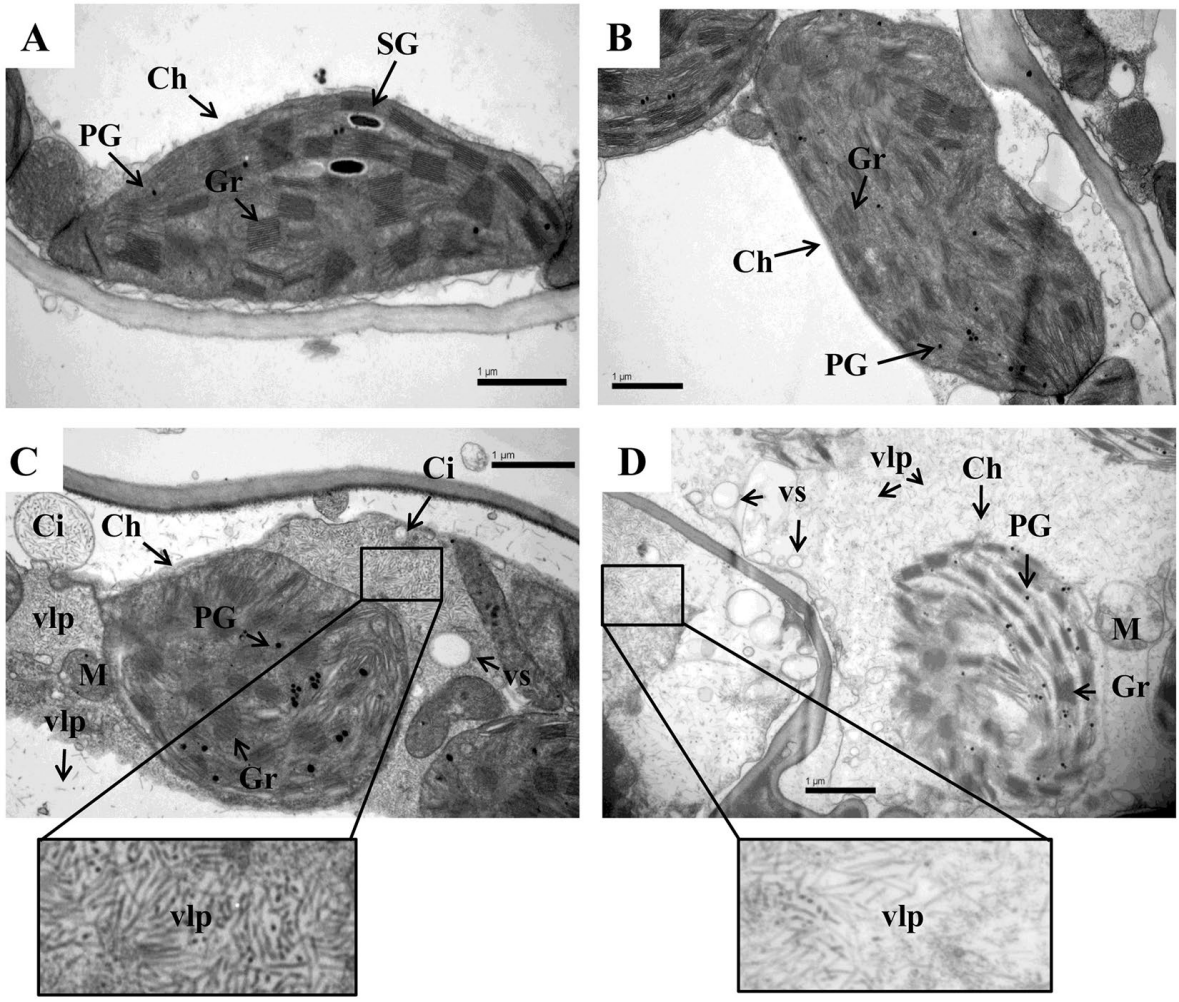
Ultrastructure of chloroplast among freeze-stressed wheat plants. (**A**) Non-silenced non-stressed; (**B**) non-silenced freeze-stressed; (**C**) freeze-stressed BSMV_0_-treated plants; (**D**) freeze-stressed BSMV_BBI_-treated plants. Labels: Ch, chloroplast; Gr, granum; M, mitochondrion; PG, plastoglobule; SG, starch grain; Ci, cytoplasmic inclusion/invagination; vlp, virus-like particles; and vs, small vesicle. Bars: 1 *μ*m.

The deleterious effects of cold stress can also manifest at the ultrastructural level^40^. In this study, BSMV_BBI_-treated plants exhibited the most serious drooping and wilting among the 3 candidate genes. Thus, these plants were sampled to observe leaf ultrastructure by TEM as well NS plants and the cold-stressed treatments. Leaf mesophyll cells of NS plants had well-developed oblong chloroplasts with regular arrangements of thylakoid lamellas in distinct grana regions (6–22 thylakoids per granum) (Figs 8A and 9A). In the FS plants and freeze-stressed BSMV_0_-treated plants, plasmolysis occurred, chloroplasts in stressed cells were distorted, and the grana stacks were disrupted (Figs 8B,C and 9B,C). Moreover, chloroplasts in the stressed viral control were abnormally rounded, vesicles of different sizes appeared, and tubular, rod-shaped virus-like particles were found within the cytoplasmic inclusions, in the cytoplasm, surrounding the chloroplasts, and clinging to the outer chloroplast membrane (Figs 8C and 9C). Rectangle/balloon-like cytoplasmic inclusions were also seen in some chloroplasts (Additional Figure S2). In addition, more plastoglobules appeared in the stressed plants (≈20 per chloroplast) than in NS plants (≈10 per chloroplast) (Figs 8B,C and 9B,C). Severe plasmolysis, vast vesicles, disrupted mitochondria, disintegrated chloroplasts, numerous tilted granal stacks, and virus-like particles were found in freeze-stressed BSMV_BBI_-treated plants (Figs 8D and 9D). These features formed a sharp contrast between stressed BSMV_BBI_-treated plants and the viral control.

## Discussion

### Comparison of iTRAQ identification of the differentially proteins in different conditions and species

As a recently developed and powerful technique, iTRAQ has been widely used to identify kinds of abiotic stress response proteins in different species. For example, iTRAQ has been successfully used to identify drought stress proteins in rice^41^, cassava^42^, maize^43, 44^, tobacco^45^, and *Brassica napus* ^46^, high salinity stress proteins in soybean^47^, cotton^48^, cucumber^49^, rice^50^, maize^51^, and *Arabidopsis thaliana* ^52^, cold stress proteins in maize^53^, Petunia^54^, and potatto^55^, and high temperature stress proteins in *Pyropia haitanensis* ^56^, rice^57^, maize^44^, and grapevine^58^, etc. The majority of these identified proteins were related to protein metabolism, carbohydrate metabolism, signal transduction, photosynthesis, transcription, cell wall and cytoskeleton metabolism, energy metabolism, membrane and transport, and stress/defense. Proteomic analyses on plant under various abiotic stress conditions revealed important information on proteins involved in the abiotic stress response^2^. For instance, higher abundance of reactive oxygen species (ROS) scavengers was detected in plants under drought, high salinity, low and high temperature stresses, and could be looked upon as a preventive measure against high oxidative damage. Moreover, all the abiotic stress conditions mentioned above were shown to induce the protein accumulated levels involved in primary metabolism (such as carbon, nitrogen, and sulfur metabolisms), indicating an enhanced energy demand during the stress conditions.

In addition, iTRAQ has also been used to identify drought, salinity, hydrogen peroxide, and Hg stress response proteins in wheat^24–27, 59^. For instance, iTRAQ was employed to identify the differentially accumulated proteins from salt-stressed wheat seedling roots. Totals of 121 stress-responsive proteins were observed, including ubiquitination-related proteins, pathogen-related proteins, transcription factors, antioxidant enzymes and membrane intrinsic protein transporters, which might work together to obtain cellular homeostasis in roots^59^. To identify the wheat protein response to Hg stress, the iTRAQ method was used to determine the proteome profiles of wheat seedlings exposed to high-Hg conditions. 249 proteins were identified with significantly altered abundance, including protein metabolism, signal transduction, stress defense, photosynthesis, carbohydrate metabolism, energy production, and transport functional groups. These findings could provide useful insights into the molecular mechanisms of Hg responses in higher plants^27^. In this study, by comparing the differentially accumulated proteins from the derivatives (UC 1110 × PI 610750), a total of 223 proteins with significantly altered abundance were identified, including protein metabolism, stress/defense, carbohydrate metabolism, lipid metabolism, sulfur metabolism, nitrogen metabolism, RNA metabolism, energy production, cell-wall metabolism, membrane and transportation, and signal transduction. The results showed that the proteome of wheat under cold stress was complex and provided an improved understanding of the molecular mechanisms involved in the tolerance of the plant to cold stress.

### Comparison of differentially accumulated proteins between CSP and CTP identified via 2-DE and iTRAQ

Using two independent cultivars showing extremely differential phenotypes to identify abiotic-related proteins could identify many unrelated proteins because many other traits’ differences maybe exist between the two surveyed cultivars. In this study, two mixed pools CSP and CTP from a F_10_ RIL population instead of only two independent cultivars/lines were used to uncover cold-responsive proteins, which can exclude the differences of genetic background between the two cultivars/lines.

One of the most important challenges in proteomics is the task of comparing 2 or more closely related proteomes to detect changes that are caused by disease, metabolic events, or experimental perturbation of a biological system^60^. While these changes have initially been measured using staining intensities via 2-DE, this approach suffers from several drawbacks, including difficulties in the analysis of several classes of proteins (e.g., membrane proteins, very large and very small proteins, and alkaline proteins) and limited sensitivity^60^. In contrast, iTRAQ overcomes the drawbacks of 2-DE. The content of peptide fragments can be reflected though adding different labels in the NH_2_- of the same peptide fragment from different samples. This ultimately allows the inference of the content of protein using software. This method possesses high efficiency, high sensitivity, quantitative accuracy, and the labeling of no more than 8 samples at one time^61^. The method has recently been widely applied to plants to understand changes in protein profiles during growth or due to environmental stimuli and stress^62, 63^. In our previous study, 23 differentially accumulated proteins between CSP and CTP of an RIL offspring were identified based on 2-DE, and one candidate gene PAP6-like was identified to potentially play an important role in conferring cold tolerance in wheat^29^. However, cold response is controlled by multiple gene loci, thus, higher flux and wider detection range iTRAQ was used to excavate more cold-responsive protein genes in this RIL population. Indeed, the advantages of iTRAQ over 2-DE are apparent regardless of the number of proteins (23 vs. 223) or functional categorizations (6 vs. 12). In total, there were 223 differentially accumulated proteins identified by iTRAQ. For instance, there were 51 metabolism-related proteins, 41 stress/defense-related proteins, 38 photosynthesis-related proteins, 15 carbohydrate metabolism-related proteins, and 4 energy production-related proteins identified by iTRAQ (Additional Table S3). This is a much higher number than the 2, 1, 9, 3, and 3-related proteins identified by 2-DE (Additional Table S3). In addition, proteins involved in lipid metabolism, sulfur metabolism, and 4 other biochemical pathways were also identified via iTRAQ. It suggested that the iTRAQ provided more comprehensive information to better understand the cold tolerance mechanisms in bread wheat. After functional verification, the identified cold-responsive proteins could be potential candidate genes in breeding for stress tolerance.

### The functional network involved in cold stress responses

Via signal transduction and regulation of gene expression, the abundance and activities of functional proteins are impacted by cold stress. Due to the significant suppression of the photosynthetic electron transport chain, ROS are readily produced in stress conditions. ROS act as signaling molecules for stress responses and also cause damage to cellular components. To counteract the harmful effects of these ROS and maintain ion homeostasis under abiotic and biotic stresses, ROS scavengers are induced in plants^2^. Of which, 14 antioxidant system members showed changes in abundance. Interestingly, only 5 antioxidant enzymes were up-regulated, e.g., superoxide dismutase [Cu-Zn] (F2DHH7), ascorbate peroxidase (J3MPG1), monodehydroascorbate reductase, chloroplastic (N1QPN2), and glutathione transferase (M8B4A4). Thioredoxin is also involved in the redox regulation by reducing disulfide bridges on target proteins^64^. One thioredoxin-like protein (W5DB05) was up-regulated by cold stress. However, most of the stress responsive proteins (9 of 13) were up-regulated. For instance, the late embryogenesis abundant protein lea14-A (M7Z4Z1) forms complexes with other macromolecules to protect the cells from stress-induced desiccation. Plant calreticulins (CRTs) appear to modulate an array of cellular responses including Ca^2+^-dependent processes, the endoplasmic reticulum (ER) chaperone response, and apoptosis^65, 66^. Expression of plant CRTs increases in response to a variety of environmental stimuli, e.g., cold, drought, and gravity^67–69^. CRT3 is induced by water stress according to the TAIR website. The up-regulated expression of CRT3 (W5CEZ6) in CSP compared with CTP implies that this calreticulin is also involved in the wheat response to cold stress.

The primary metabolisms, such as carbon, nitrogen, sulfur, and energy metabolisms, need to be modulated in response to cold stress. Our results show that 15 carbohydrate metabolic enzymes exhibited changes in abundance in CSP compared with CTP (Additional Table S2). Within this group, the levels of most of the proteins related to glycolysis (5 of 7, 71.4%) increased. For example, glyceraldehyde-3-phosphate dehydrogenase (O22387), glyceraldehyde-3-phosphate dehydrogenase chloroplastic-like (J3LJK2), and phosphoenolpyruvate carboxylase (M0XEC5). Their up-regulation might help to produce more energy needed in cold defense processes. In comparison, 6-phosphogluconate dehydrogenase, decarboxylating-like isoform 1 (I1H2R0) and putative 6-phosphogluconolactonase 4, and chloroplastic (W5FQX8) involved in the pentose phosphate pathway (PPP) were down-regulated. Two Ferredoxin-nitrite reductases (NiR) (M0W559 and W5GWJ4) related to nitrogen metabolism were also up-regulated by cold stress. NiR catalyzes the reduction of nitrite to ammonium in the second step of the nitrate-assimilation pathway. NiR changes in etiolated rice seedlings were induced by nitrate and light^70^. Two sulfur metabolism-related enzymes, putative plastidic cysteine synthase (W5FLB0) and S-adenosylmethionine synthetase 1 (A0A096UUP6), were up-regulated by cold stress. Cysteine synthase (CS) catalyzes the final reaction in the cysteine biosynthetic pathway. This reaction is also a key limiting step in the production of glutathione, a thiol implicated in resistance to biotic and abiotic stresses. A significant accumulation of CS under cold stress has been reported in rice, *Lolium perenne*, and wheat^13, 16, 71, 72^. S-adenosylmethionine synthetase catalyzes the biosynthesis of S-adenosyl-L-methionine, a precursor for the biosynthesis of polyamines and ethylene. A close correlation between cold tolerance and polyamine accumulation level in rice under cold stress has been reported. The up-regulated expression of ATP synthase beta subunit (A9L9Z4), ATP synthase CF1 beta subunit (A0A0F6NQY1), and ATP synthase subunit a (chloroplast) (A0A0E0EPX3) revealed that energy metabolism was altered under cold stress. ATP synthases are membrane-bound enzyme complexes/ion transporters that connect ATP synthesis and/or hydrolysis with the transport of protons across a membrane. These enzymes play critical roles in the removal of damaged proteins and in the control of some key cellular components, combining peptidase and chaperone activities^73^. Overexpression of the ATP synthase gene in transgenic *Arabidopsis* caused an increased resistance to salt, drought, and cold stresses^74^. The up-regulation of carbohydrate catabolism, nitrogen metabolism, sulfur metabolism, and energy pathway-related proteins in CSP may contribute to stabilizing the cellular osmotic pressure. The up-regulation was a result of the increased energy demand in plants exposed to cold stress.

### Cold stress enhances protein degradation

Proteomic analysis showed that several proteins were partially degraded by cold stress, especially the components of the photosynthesis apparatus, cell-wall metabolism, membrane and transportation, and lipid metabolism (Additional Table S2). Similarly, the results from a diploid wild wheat proteome under cold stress showed that several proteins were down-regulated^16^. These results indicated that LT enhanced protein degradation. Photosynthesis is thought to be one of the first processes influenced by cold stress^14^. One typical example of a photosynthesis molecule is ribulose-1,5-bisphosphate carboxylase/oxygenase (Rubisco), of which 9 subunits or subunit fragments were identified in this study. Most of these (6, representing 66.7%) were down-regulated. The degradation of Rubisco has also been reported in cold stressed rice and wheat proteomic studies^13, 14, 16, 71, 75^. Moreover, our results also provide evidence for the degradation of other photosynthetic proteins such as the oxygen evolving complex of photosystem II subunit (A3BKU8), carbonic anhydrase (F2DZH9 and B4F9E2), chlorophyll a-b binding protein, chloroplastic (M0 × 0F4 and M0ZEV8), chlorophyll a-b binding protein 1B, and chloroplastic (M8CA60). These results suggest that the photosynthesis apparatus is susceptible to cold stress. This may be one of the major reasons for decreased net photosynthetic rate under cold stress conditions. Another typical example of cell-wall metabolism is fasciclin-like arabinogalactan proteins (FLAs), which are a subclass of cell wall glycoprotein family-arabinogalactan proteins (AGPs). Fasciclin-like arabinogalactan protein 11-like (M8BBJ1) and fasciclin-like arabinogalactan protein 7-like (A0A0A9EJ37) were identified in our study. Indeed, FLAs are implicated in cell wall biosynthesis, cell wall remodeling, and signaling. FLAs have also been reported in the response to salt stress^76, 77^. Furthermore, our results provide the first evidence for the degradation of these 2 FLAs by cold stress.

### Wheat silenced for the 3 candidate protein genes under cold stress using VIGS

Hsp90s are ubiquitous molecular chaperones in the cells of eukaryotes and eubacteria. They play key roles in signal transduction, protein folding, protein degradation, growth and developmental programs, and responses to environmental stimuli^78^. Several studies have shown growth inhibition and abnormalities in T-DNA knockout lines lacking any of the 4 cytosolic *Arabidopsis thaliana* AtHsp90 genes^79^. The silencing of cytosolic Hsp90 expression in *Nicotiana benthamiana* and soybean resulted in stunted and deformed leaf phenotypes^80–82^. In our study, the identified Hsp90 shared 99% identity with TaHsp90.2. Moreover, it has been verified that the suppression of the TaHsp90.2 gene via VIGS compromised the hypersensitive resistance response of the wheat variety to stripe rust fungus^83^. Our study confirmed the role of Hsp90 in the response to cold stress in wheat. Protease inhibitors respond to a number of cellular physiological processes by regulating protease activity^84^. BBI-type protease inhibitors are common in both developing seeds and wounded plant tissue. In addition to its well-studied role in defense against pathogenesis, it may also be involved in abiotic stress response. For instance, Shitan *et al*.^85^ showed that BBI was responsible for tolerance to excess cadmium in yeast. Three wheat WIP1-like genes (wali3, wali5, and wali6) were induced by wounding or by the imposition of aluminum ions or toxic metal stress^86, 87^. WRSI5 is induced by salt stress, drought, Al^3+^, and H_2_O_2_ stress. Furthermore, the overexpression of WRSI5 improved the salt tolerance of *A. Thaliana* ^84^. In our study, one BBI was successfully identified, which is induced by cold stress. Furthermore, our results represent the first verification of the role of this gene in wheat cold tolerance. Cold acclimation is triggered by the exposure of plants to low but nonfreezing temperatures for certain periods of time. During this process, plants exhibit dramatic alterations in their gene expression profiles. These changes include the induction of an array of cold-responsive (Cor) genes^7^. Wheat and barley possess a small family of Cor genes including Wcs19, Wcor14, and Bcor14b^88–90^, all of which encode chloroplast-targeted COR proteins analogous to the *Arabidopsis* protein COR15a^91^. The wheat Cor gene Wcor15 was isolated in 2003, and it encodes a chloroplast-targeted protein and shares LT specificity with the barley and wheat Cor genes. The expression of Wcor15 was specifically induced by LT in wheat leaves, and light illumination markedly increases the steady-state level of its transcripts^92^. Furthermore, transgenic lines expressing the Wcor15-GFP fusion gene showed a significantly improved level of freezing tolerance compared with wild-type tobacco plants^92^. In our study, REP14 has a 95% identity with Wcor15, and the abundance of this protein was increased in CTP compared with CSP after over-wintering. The qRT-PCR results showed that REP14 was also induced under cold stress and the role of this gene in wheat cold tolerance needs to be further studied.

VIGS was successfully used in monocotyledonous barley using BSMV^93^. Indeed, the development of VIGS for functional genomics in monocots is significant because of the difficulty in applying other loss-of-function approaches requiring transformation to these species^37^. Since this technology was first successfully used in bread wheat^35^, VIGS has been widely used for analysis of gene function for resistance of wheat pathogen and wheat aphid as well as water stress response genes^35–37, 39, 83, 94^. Moreover, we successfully applied VIGS on the functional analysis of cold-responsive genes in wheat. In this study, VIGS was also carried out for the functional validation of three candidate cold-responsive genes Hsp90, BBI, and REP14. Additionally, stress is associated with structural cell damage and reduced moisture content in plants^15^; these changes may lead to plant lodging or leaves wilting^95^. In this study, leaves of the freeze-stressed BSMV_Hsp90_, BSMV_BBI_, and BSMV_REP14_-treated plants showed distinctly more severe droop and wilt than the freeze-stressed viral controls. This phenotypic result was confirmed by markedly increased rates of relative electrolyte leakage but decreased RWC in the freeze-stressed BSMV_HSP90_, BSMV_BBI_, and BSMV_REP14_-treated plants, these results indicate important roles of Hsp90, BBI, and REP14 in confering water and low-temeprature stress in wheat.

Until recently, TEM images of thin sections of BSMV-infected leaves were only examined in epidermal cells of *Nicotiana benthamiana* and barley^96^. This is the first time we have conducted TEM for the leaves of BSMV-infected wheat. Chloroplasts are the first and most severely impacted organelle under cold injury. It is typical that plasma membranes invaginate (plasmolyse), the shape and size of the chloroplasts change, thylakoids become swollen and distorted, and numerous plastoglobules accumulate under cold stress^97, 98^. This study was consistent with these results, and the ultrastructural morphological injuries were apparent in wheat leaves under freezing stress, especially in gene silenced plants. It was found that freeze-stressed BSMV_0_-treated plants suffered less serious injuries in mesophyll cells than freeze-stressed BSMV_BBI_-treated plants. Furthermore, freeze-stressed BSMV_0_-treated plants exhibited better-developed granal stacks and less invagination of plasma membrane than freeze-stressed BSMV_BBI_-treated plants. For instance, chloroplast disintegration was observed in freeze-stressed BSMV_BBI_-treated plants but not in BSMV_0_-treated plants. In addition, TEM images of abnormally rounded chloroplasts, cytoplasmic inclusions, and their association with virus-like particles obtained by using the BSMV are similar to images of thin sections of BSMV-infected *Nicotiana benthamiana* and barley^96^. The difference is that there were fewer chloroplasts which contained membrane-bound cytoplasmic inclusions (virus-like particles) in wheat than in *Nicotiana benthamiana* and barley. The reasons contributing to the differences could possibly owing to virus-infected period and duration, experimental materials, and treatments.

## Electronic supplementary material

Additional Figures and Tables

Additional file 1

Additional file 2

Additional file 3

Additional file 4

Additional file 5
