# Supplementary material for: iTRAQ and virus-induced gene silencing revealed three proteins involved in cold response in bread wheat
Source: Sci Rep. 2017 Aug 8;7:7524. doi: 10.1038/s41598-017-08069-9 (PMC5548720; doi:10.1038/s41598-017-08069-9)
Supplement: Supplementary file 1 — Additional Figures and Tables [file 41598_2017_8069_MOESM1_ESM.pdf]

Supplementary information

iTRAQ and virus-induced gene silencing revealed three proteins involved in cold response in bread wheat

Zhang Ning, Zhang Lingran, Zhao Lei, Ren Yan, Cui Dangqun, Chen Jianhui, Wang Yongyan, Yu Pengbo, Chen Feng*

Agronomy College/National Key Laboratory of Wheat and Maize Crop Science/Collaborative Innovation Center of Henan Grain Crops, Henan Agricultural University, Zhengzhou 450002, China

* Corresponding authors: F. Chen (chf0088@163.com)

Agronomy College, Henan Agricultural University, 95 Wenhua Road, Zhengzhou 450002, P. R. China

Phone: +86-371-63558537

Additional Figure S1. The success rate of the plants inoculated with different BSMVs

(A) Values are means (±SE) of three independent biological replicates (20 seedlings/group). Bars represent standard errors of triplicate experiments.

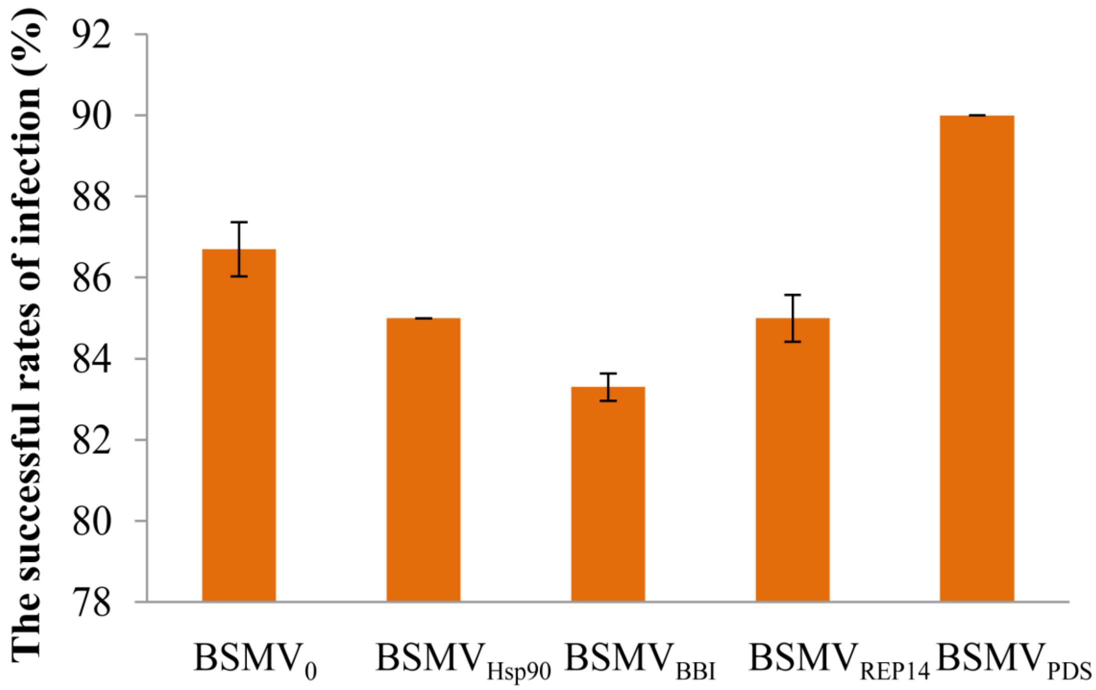

Additional Figure S2. TEM images of abnormal chloroplasts with cytoplasmic inclusions containing virus-like particles of thin sections of virus-infected leaves.

(A)

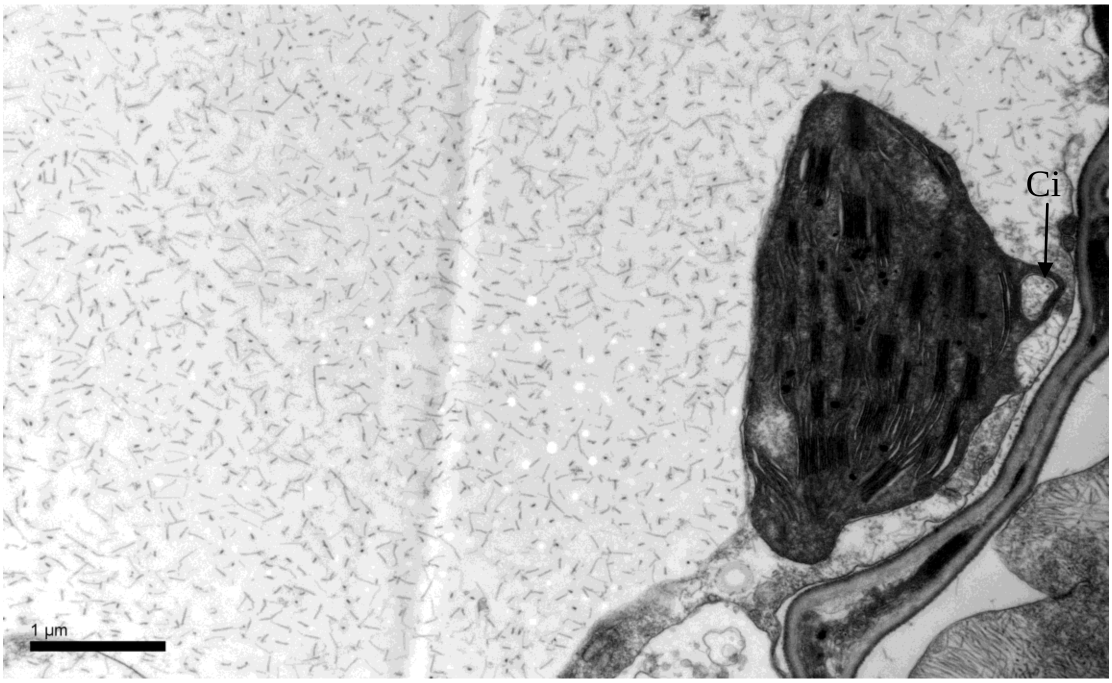

(B)

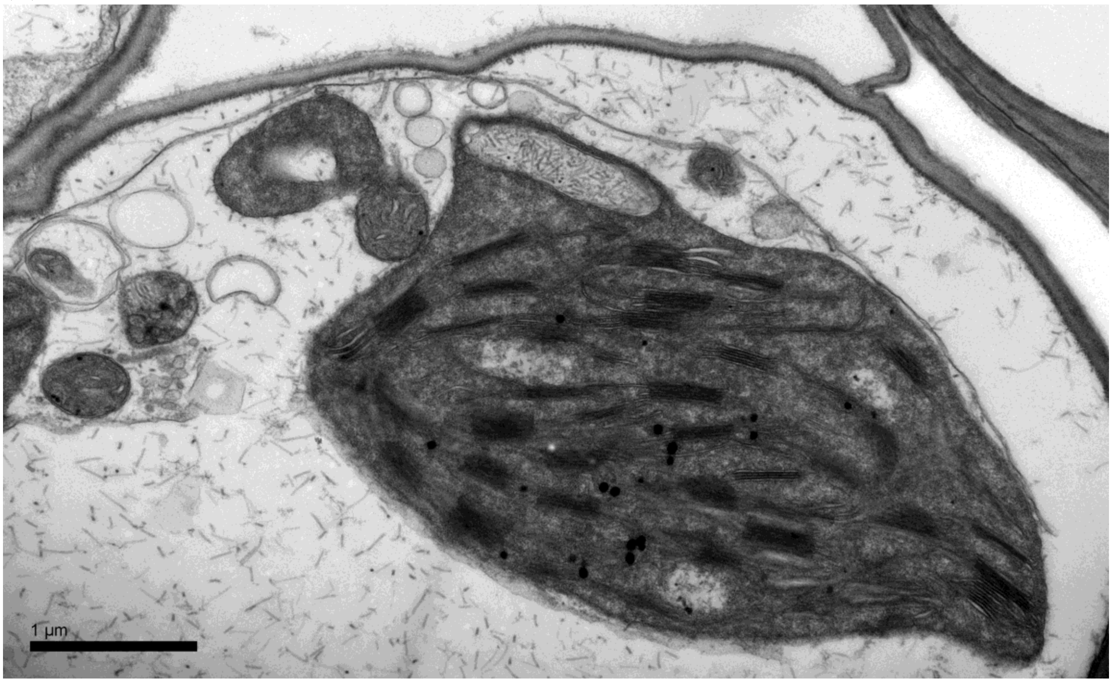

Additional Table S1. Primer sequences used in qRT-PCR of the 20 cold-responsive protein genes and VIGS of the three candidate genes

Accession no.[a]	Forward primer	Reverse primer	Produce length (bp)
M7Z4Z1	TCCTTCAACCTCAAGTGCGG	AAGTTGCCGATGATGGGGAG	218
M8BTT2	GTCGTTGCAGCACTTCCATG	ATCGCTCTTCCTTGTCCAGC	260
F2CQU0	AAGTGTGTCAACTGCGATGG	CATCCTTGAACTCCCTGCGA	100
M7YSY0	TGTTCGTGGCCTTGCATACT	CTAGATGCTGGCCCCTCAAG	264
Q84ZY0	TACAGCCGCCATTGTGAAGT	TGAAAGTTCGACCCCAGACG	145
M7YJJ9	TGAGGACCCTGAACGCCAA	TGTTGATGGGGAAAGAGACGG	150
A0A0F6NQY1	CCAAATGAATGAACCGCCGG	TGTGCAAATGTTGTGGCAGG	324
O22387	CATCGCCATCAACAACACCG	ATGACGAGGTTGATGCCGAG	198
F2CU34	CTGAGGAGGGCAAGGTTGAG	CCAGTCGTTTGTCAGGCTCT	188
M8BGU7	GATCGACCTGCCCTACTCCT	TCGTCGAGGAAGCTCTGGT	102
Q8S385	AGCGAAGTGGACGACAAGG	CTTGTCCGTGATGCCCTGT	105
M8C3D9	TGATCACCCCGGACCTGTT	GCGGTGGTCTTGTTCTTCAC	103
N1QPN2	TGTTTACGCCTTCCCTTGCT	CACCCTTCCATCAGAGCCAG	113
B4ESE2	CTTTGCTGTCCTGCTCGGTA	GCTCCCAGATTCACAGGCTT	241
F2DHH7	CAACGGCTGCATGTCAACTG	CATGTCCACCCTTGCCAAGA	233
O80370	GGAGGTTTAAGGGGTCCACG	TCACCTTGACTACACCACGC	123
P46285	AAGGGCATCTTCACGAACGT	TGATCTCGTTCTTGGAGCCG	196
A0ST49	CCGATATTCAACAACGCGTCC	TGGCGGTAGATGACGGAGA	167
I1GTJ6	TCGAGGAATGGCTCAAGACG	GTTCTCGGCGGTGTTAAACG	170
M8BYV1	GTGCTACACCCGATCCACAA	GAGTTCGTTGGGGAGAGCAA	228
β-actin	GTTCCAATCTATGAGGGATACACGC	GAACCTCCACTGAGAACAACATTACC	422
V-REP14	GAGTTGAAGAAGGGCGTGGC	CTTGTCCGTGATGCCCTGT	189
V-BBI	CATCGACGGTGCTCTTCATG	CATCGACGGTGCTCTTCATG	198
V-Hsp90	CTGAGGAGGGCAAGGTTGAG	CCAGTCGTTTGTCAGGCTCT	188
V-PDS	TCACAAGTAGCAGCGTCCA	GGTTTACTGGGGCGTTCACT	211

[a] Accession no.: accession number in Uniprot database corresponds to the iTRAQ results, correspondingly are the same as shown in Table S2. (except for *β*-actin and PDS)

Additional Table S2. Functional classifications of the differentially accumulated proteins identified by iTRAQ between CSP and CTP

Spot no.[a]	Proteins	MV[b]	p*I*[c]	Coverage (%)	Nup.[d]	Ratio[e]	P-value	Plant species
**Unknown**								
M8BZI9	Hypothetical protein F775_07291	42.34	9.98	8.78	1	0.60	0.00585264	*A. tauschii*
A0A096UU59	Hypothetical protein F775_29027	9.85	9.69	20	1	1.27	0.0315009	*A. tauschii*
M8BQ48	Hypothetical protein F775_32603	18.49	9.57	18.64	1	0.73	0.0316062	*A.tauschii*
M7Z4H8	Hypothetical protein TRIUR3_00731	8.87	8.51	33.33	2	1.21	0.0388632	*T. urartu*
W5DP76	Hypothetical protein TRIUR3_04889	9.17	9.32	14.12	1	0.73	0.00431867	*T. urartu*
M7ZY44	Hypothetical protein TRIUR3_10983	16.22	8.69	15.03	2	0.72	0.00704315	*T. urartu*
W5FX26	Hypothetical protein TRIUR3_12762	11.37	8.31	14.85	1	1.27	0.0135276	*T. urartu*
W5E2P5	Hypothetical protein TRIUR3_12806	19.44	10.30	6.04	1	0.56	0.00672056	*T. urartu*
I1GR21	Hypothetical protein TRIUR3_14708	38.36	7.01	3.48	1	0.72	0.00587336	*T. urartu*
M7XL46	Hypothetical protein TRIUR3_33418	14.56	8.19	14.29	1	1.44	0.0150124	*T. urartu*
F2CXA5	Predicted protein	47.55	4.64	20.76	1	1.42	0.0413252	*H. vulgare*
A0A077RU93	Predicted protein	34.15	8.75	13.03	3	1.21	0.0309323	*H. vulgare*
T1NNC4	Predicted protein	11.45	9.98	12.26	1	0.79	0.0245215	*H. vulgare*
M0X1S6	Predicted protein	11.66	9.63	12.61	1	0.67	0.0221063	*H. vulgare*
F2DA26	Predicted protein	35.63	8.44	9.79	2	0.80	0.0164073	*H. vulgare*
I1HN58	Uncharacterized protein LOC100834407	9.50	9.94	10.11	1	1.43	0.0166079	*B. distachyon*
A0A096ULD8	Hypothetical protein TRIUR3_11917	41.33	5.49	9.52	2	0.76	0.0203294	*T. urartu*
W5BQ56	Predicted protein	38.74	5.81	29.44	1	0.70	0.00281769	*H. vulgare*
M8AZ52	Hypothetical protein F775_07046	24.28	7.47	20.18	3	0.73	0.0212763	*A. tauschii*
M8BXT4	Hypothetical protein F775_30291	23.28	8.91	27.44	2	0.78	0.0180222	*A. tauschii*
N1QQE6	Hypothetical protein F775_32959	7.75	4.06	17.81	1	0.68	0.0146192	*A. tauschii*
M7ZY79	Hypothetical protein TRIUR3_26332	85.64	7.85	8.66	1	1.23	0.000330028	*T. urartu*

Spot no.[a]	Proteins	MV[b]	p*I*[c]	Coverage (%)	Nup.[d]	Ratio[e]	P-value	Plant species
**Proteins metabolism**								
M0VC57	30S ribosomal protein chloroplastic	10.70	10.11	31.91	1	0.53	0.00806831	*H. vulgare*
I1GY66	30S ribosomal protein S31, chloroplastic-like	13.54	9.45	7.2	1	0.77	0.0274252	*B. distachyon*
W5AY25	plastid-specific 30S ribosomal protein 2	19.12	5.14	50.87	1	1.41	0.0241303	*A. tauschii*
M7Z315	40S ribosomal protein S13-1	16.89	10.36	7.95	1	0.82	0.0231845	*T. urartu*
K3ZXP8	40S ribosomal protein S15-like	17.29	10.20	20.26	2	0.81	0.0163879	*S. italica*
W5AH72	40S ribosomal protein S17-4	13.67	9.52	53.33	1	0.64	0.0029642	*A. tauschii*
W5A5L6	40S ribosomal protein S17-4	16.48	10.21	44.76	1	1.35	0.00145439	*T. urartu*
M7YRN1	40S ribosomal protein S20	14.10	9.44	18.9	1	0.75	0.046111	*T. aestivum*
F2EA93	40S ribosomal protein S20	14.05	9.51	18.9	1	0.75	0.0231379	*H. vulgare*
W5EUP3	40S ribosomal protein S26-1	14.28	10.70	27.48	1	0.82	0.0176856	*A. tauschii*
F2CV88	40S ribosomal protein S26-2-like	15.68	11.03	27.08	1	0.56	0.0073136	*H. vulgare*
I1I8W1	60S ribosomal protein L17-2-like	18.91	10.14	9.7	1	0.81	0.0216686	*B. distachyon*
W5BMT1	60S ribosomal protein L19-2	7.48	10.62	36.76	1	1.47	0.011655	*A. tauschii*
M8AWE2	60S ribosomal protein L22-2	14.44	9.55	29.77	2	0.76	0.00601131	*A. tauschii*
R7W4C5	60S ribosomal protein L27-3	14.40	10.29	18.6	1	0.70	0.00286792	*A. tauschii*
W5BNZ6	60S ribosomal protein L27a-3	15.98	10.67	35.17	2	0.80	0.00164843	*T. urartu*
A0A0A9QPU7	60S ribosomal protein L36-2-like	8.56	11.11	13.7	1	1.21	0.0216967	*B. distachyon*
R7VYR3	Histone H2A	13.07	10.68	25.2	1	0.64	0.0347828	*A. tauschii*
B6TFY8	Histone H4	11.42	11.47	50.49	1	0.22	0.000362137	*Z. mays*
Q9FYV3	Elongation factor	49.29	8.95	29.02	1	0.32	0.000184401	*S. officinarum*
W5G125	Elongation factor 2	93.87	6.16	33.81	1	0.82	0.0459369	*T. urartu*
R7W2H4	Elongation factor ts	100.90	5.01	32.02	1	0.67	0.00423554	*A. tauschii*

Spot no.[a]	Proteins	MV[b]	p*I*[c]	Coverage (%)	Nup.[d]	Ratio[e]	P-value	Plant species
C5X4S1	Elongation factor Ts family protein	102.05	5.26	7.47	1	1.23	0.00921989	*S. bicolor*
F2DJN3	Proteasomal ubiquitin receptor ADRM1	33.49	4.55	15.03	2	0.80	0.0385949	*H. vulgare*
A0A088CDA2	Leukotriene A-4 hydrolase homolog	12.32	4.32	12.84	1	0.74	0.0397254	*B. distachyon*
W5B7Z6	Adenosylhomocysteinase	53.21	5.80	31.34	1	1.26	0.019354	*A. tauschii*
I1J202	Aminomethyltransferase, mitochondrial-like	43.81	8.57	31.87	2	1.21	0.0129526	*B. distachyon*
M8AT56	Ankyrin repeat domain protein expressed	29.88	4.56	35.19	1	1.38	0.0139324	*A. tauschii*
N1QZ60	Aspartic proteinase nepenthesin-1	48.67	6.52	2.88	1	0.79	0.0418379	*A. tauschii*
W5GPA3	Eukaryotic translation initiation factor 3 subunit F-like	22.29	5.25	12.44	1	1.40	0.0147752	*H. vulgare*
M8CRB5	F-box protein SKIP8	27.19	5.27	13.1	3	0.72	0.0247892	*A. tauschii*
M7YSY0	Histidyl-tRNA synthetase	45.70	6.00	7.44	2	0.78	0.0389197	*T. urartu*
J3L8Q1	Hydroxypyruvate reductase	42.02	7.40	36.53	1	1.24	0.00169442	*O. sativa*
M7YX26	Methylthioadenosine/S-adenosylhomocysteine nucleosidase	37.09	8.66	6.94	2	0.79	0.0357827	*T. urartu*
W5FEI2	Aspartate carbamoyltransferase 3, chloroplastic	16.65	7.44	8.05	1	0.79	0.0067484	*A. tauschii*
A0A077S078	ATP-dependent Clp protease proteolytic subunit 3, chloroplastic-like	29.86	9.20	5.54	1	0.83	0.0103021	*B. distachyon*
F2CSC3	ATP-dependent Clp protease proteolytic subunit-related protein chloroplastic	30.92	9.26	10.28	2	0.74	0.000917406	*H. vulgare*
W5GYP6	Histidinol-phosphate chloroplastic-like	40.60	6.93	4.67	1	0.79	0.0456801	*H. vulgare*
F2E066	Chloroplast nucleoid DNA-binding protein CND41	18.90	8.59	6.04	1	0.67	0.0127066	*H. vulgare*
M8C184	Mitochondrial glycoprotein	24.07	4.37	15.49	1	0.78	0.0208519	*A. tauschii*
W5FWL8	TPA: histone acetyltransferase HPA2 and	16.94	10.39	8.23	1	1.22	0.00360847	*A. tauschii*

Spot no.[a]	Proteins	MV[b]	p*I*[c]	Coverage (%)	Nup.[d]	Ratio[e]	P-value	Plant species
	acetyltransferase							
B9VTA3	Ubiquitin fusion degradation 1 protein	35.47	7.44	4.95	1	0.73	0.00406736	*T. aestivum*
M8AKZ8	WD-40 repeat	130.30	6.15	4.03	2	1.26	0.0260271	*A. tauschii*
M0yi51	Translation elongation initiation factor family protein	34.66	8.18	19.68	1	1.27	0.0431456	*A. Tauschii*
W5AN26	Dihydrodipicolinate reductase	25.57	8.69	9.28	1	0.78	0.044142	*H. vulgare*
F2CQU0	Protein disulfide-isomerase LQY1-like	16.79	7.50	13.38	1	1.23	0.0392989	*H. vulgare*
M7ZVH9	Protease Do-like 5, chloroplastic	27.88	5.82	9.09	2	0.55	0.0000739	*T. urartu*
W5HUN1	Thylakoid membrane protein slr0575-like	12.05	8.44	21.15	1	1.22	0.0332807	*H. vulgare*
W5FWD2	Dihydrolipoamide acetyltransferase	31.39	9.50	15.33	1	0.66	0.023494	*H. vulgare*
W5FH48	Dihydrolipoamide acetyltransferase	36.87	8.92	12.57	1	0.79	0.0227194	*S. bicolor*
I1HK18	Nascent polypeptide-associated complex subunit alpha-like protein 1	22.24	4.40	21.57	1	0.78	0.00496917	*B. distachyon*
W5GKA6	HOP	34.05	7.30	14.97	1	0.43	0.000818621	*T. aestivum*
F2CU34	Heat shock protein 90	80.36	5.06	22.71	1	1.25	0.0201855	*H. vulgare*
W4ZRZ2	Sorting and assembly machinery component 50-A-like protein	56.38	5.92	2.89	1	0.83	0.0192565	*A. tauschii*
**Sulfur metabolism**								
A0A096UUP6	S-adenosylmethionine synthase 1	51.90	5.81	20.51	1	1.23	0.0388076	*T. urartu*
W5FLB0	Cysteine synthase	46.18	8.84	32.95	2	1.30	0.000457825	*T. aestivum*
**Nitrogen metabolism**								
M0W559	Ferredoxin-nitrite reductase precursor	56.22	5.91	23.91	1	1.21	0.0386021	*H. vulgare*
W5GWJ4	Ferredoxin-nitrite reductase precursor	56.23	6.20	31.62	2	1.28	0.0137346	*T. aestivum*

Spot no.[a]	Proteins	MV[b]	p*I*[c]	Coverage (%)	Nup.[d]	Ratio[e]	P-value	Plant species
**Stress/defense**								
M7ZFY1	Peroxidase 1	34.72	7.83	9.37	1	0.82	0.0214046	*T. urartu*
W5I0G1	Peroxidase 1	16.41	6.51	26.11	2	0.65	0.0155338	*T. urartu*
W5ETT8	Oxalate oxidase GF-2.8	7.13	9.54	15.15	1	0.53	0.00271372	*A. tauschii*
W5B840	Peroxidase 12	34.16	5.90	19.88	1	0.74	0.0194406	*H. vulgare*
M8C3D9	Peroxidase 12	30.66	5.49	21.84	1	0.53	0.0167645	*A. tauschii*
T1LHN5	Peroxidase 70	26.63	5.62	21.51	1	0.53	0.00686877	*A. tauschii*
W5BZ90	Lipoxygenase 1	54.38	7.61	26.05	1	0.43	0.00423582	*T. urartu*
M8BYV1	Putative lipoxygenase 4	38.07	8.03	16.23	1	0.46	0.000348988	*A. tauschii*
T1LSZ4	Lipoxygenase 2.1, chloroplastic	104.05	6.00	6.02	1	0.73	0.00562947	*A. tauschii*
K3XV98	Lipoxygenase 2.1, chloroplastic-like	95.29	6.04	3.11	1	0.70	0.0282982	*S. italica*
M7ZI22	NAD(P)H-quinone oxidoreductase subunit H, chloroplastic	54.82	9.50	11.94	1	0.75	0.0220624	*T. urartu*
M0W2C4	NADH dehydrogenase [ubiquinone] iron-sulfur protein mitochondrial-like	75.19	6.42	21.18	1	0.68	0.0362073	*H. vulgare*
W5H151	Alcohol dehydrogenase class-3-like	40.63	6.81	25.72	1	1.23	0.0209683	*B. distachyon*
O80370	VER2	32.42	7.18	23	5	0.39	0.00621467	*T. aestivum*
W5CEZ6	Calreticulin-3	44.97	6.76	8.05	2	1.21	0.011462	*T. urartu*
K7UNK1	Mitochondrial uncoupling protein 3	15.55	9.26	11.19	1	1.23	0.00398343	*Z. mays*
F2DAA1	Cold-regulated protein	17.60	5.06	14.02	2	0.75	0.0348304	*H. vulgare*
Q8S385	REP14	14.73	6.19	48.63	1	2.30	0.0172345	*S. cereale*
M7Z4Z1	Late embryogenesis abundant protein lea14-A	17.82	5.21	6.75	1	1.36	0.0432786	*T. urartu*
W5EEV4	Putative inactive purple acid phosphatase 27	69.08	6.46	7.94	4	0.79	0.00581916	*A. tauschii*

Spot no.[a]	Proteins	MV[b]	p*I*[c]	Coverage (%)	Nup.[d]	Ratio[e]	P-value	Plant species
F2DHH7	Superoxide dismutase [Cu-Zn]	15.09	6.28	31.58	1	1.23	0.0073188	*H. vulgare*
I1GRB8	Superoxide dismutase [Cu-Zn] 4A-like	15.14	6.05	8.55	1	0.46	0.00249884	*B. distachyon*
J3MPG1	Ascorbate peroxidase	27.14	5.36	22	1	1.30	0.0365614	*O. sativa*
W5GFX3	Peroxiredoxin-2E- chloroplastic-like	23.26	8.75	59.29	1	0.81	0.0102177	*H. vulgare*
N1QPN2	Monodehydroascorbate reductase, chloroplastic	52.41	7.71	14.97	1	1.24	0.0127119	*A. tauschii*
Q84UH6	Dehydroascorbate reductase	23.34	6.29	61.32	2	0.80	0.0113331	*T. aestivum*
A0ST49	Polyphenol oxidase	14.78	5.15	10	1	1.41	0.00383159	*T. aestivum*
M8B4A4	Glutathione transferase	25.18	5.34	7.3	1	1.22	0.0176968	*T. aestivum*
R7WCP0	Probable glutathione S-transferase GSTU6-like	25.70	5.58	24.07	2	0.79	0.0372786	*A. tauschii*
D8L9S2	Glutamate decarboxylase, putative, expressed	54.07	5.78	28.34	1	1.32	0.00620892	*T. aestivum*
F1DKC1	Catalase	56.85	7.08	8.3	2	0.80	0.0383464	*T. aestivum*
W5DB05	Thioredoxin-like protein	8.43	5.10	39.19	1	1.37	0.0334654	*A. tauschii*
W5EC03	Rubredoxin family expressed	16.86	9.96	15.69	1	1.24	0.0284005	*H. vulgare*
M8BHR2	Monothiol glutaredoxin-S12, chloroplastic	30.55	7.93	3.86	1	0.80	0.0032993	*A. tauschii*
B4ESE2	Legumain	53.06	6.42	2.69	1	1.48	0.0106641	*H. vulgare*
M7YVE8	Bowman-Birk type protease inhibitor	9.59	8.35	18.89	1	1.28	0.00711271	*T. aestivum*
M0XIW2	Complex I intermediate-associated protein 30	69.20	9.01	18.97	1	1.62	0.035929	*B. distachyon*
A0A096UQG2	Acid phosphatase 1	14.49	9.74	8.4	1	0.66	0.0353554	*T. urartu*
**Carbohydrate catabolism**								
O22387	Glyceraldehyde-3-phosphate dehydrogenase	28.31	9.57	42.91	1	1.33	0.0211264	*O. sativa*
J3LJK2	Glyceraldehyde-3-phosphate dehydrogenase chloroplastic-like	49.71	7.91	34.26	3	1.25	0.00375161	*O. sativa*
C1JYE0	3-phosphoglycerate kinase, partial	31.34	5.01	44.63	1	0.48	0.00628541	*D. villosum*

Spot no.[a]	Proteins	MV[b]	p*I*[c]	Coverage (%)	Nup.[d]	Ratio[e]	P-value	Plant species
I1H2R0	6-phosphogluconate dehydrogenase, decarboxylating-like isoform 1	53.85	6.18	19.36	2	0.57	0.0238126	*B. distachyon*
W5E8Y1	Beta-glucosidase 8	52.74	8.19	3.18	1	0.68	0.0207587	*T. urartu*
A0A0D9WD69	Fructose-6-phosphate-2-kinase/fructose-2,6-bisphosphatase	86.74	6.68	9.36	1	1.31	0.0273614	*O. sativa*
M8C3S5	Malate dehydrogenase (NADP), chloroplastic	41.98	5.53	25.91	2	0.77	0.0480375	*A. tauschii*
M0XEC5	Phosphoenolpyruvate carboxylase	113.31	5.48	22.13	1	1.40	0.0020871	*H. vulgare*
M7ZKL3	Phosphoenolpyruvate carboxylase 2	108.40	6.10	16.95	12	1.20	0.0129287	*T. urartu*
H9ZX57	Phosphoglycerate kinase	31.42	5.01	67.11	1	0.48	0.00534659	*T. timopheevii*
W5FQX8	Putative 6-phosphogluconolactonase 4, chloroplastic	23.00	5.85	11.06	2	0.79	0.0485737	*A. tauschii*
W5I774	Sucrose synthase 1	92.34	6.09	16.21	1	0.75	0.00174838	*A. tauschii*
Q84ZY0	Sucrose-phosphatase	47.14	6.37	14.93	5	1.24	0.0369452	*A. speltoides*
I1HWP9	3-isopropylmalate dehydratase-like	55.85	7.43	18.02	1	0.34	0.0038624	*B. distachyon*
M0Y0Y6	Cell wall invertase 1	47.35	8.16	5.87	2	0.80	0.0148794	*H. vulgare*
**Photosynthesis**								
K7U064	Photosystem I subunit VII	8.72	6.47	55	1	1.38	0.0221731	*Z. mays*
A0A089N372	Photosystem I subunit VII	8.88	6.92	81.48	1	0.82	0.0288271	*O. sativa*
F2CQY2	LHCI-680, photosystem I antenna protein	27.28	5.94	29.02	4	1.29	0.0253433	*H. vulgare*
Q6XW17	Photosystem II polypeptide	10.19	8.65	54.55	5	1.23	0.0197746	*T. aestivum*
W5D4R0	Photosystem II subunit	28.29	8.82	31.25	1	1.31	0.022308	*H. vulgare*
Q66MG6	Ribulose-1,5-bisphosphate carboxylase/oxygenase large subunit	49.74	6.92	37.42	1	0.81	0.0159074	*S. cultivar*

Spot no.[a]	Proteins	MV[b]	p*I*[c]	Coverage (%)	Nup.[d]	Ratio[e]	P-value	Plant species
U5IM61	Ribulose-1,5-bisphosphate carboxylase/oxygenase large subunit, partial (chloroplast)	21.76	6.57	29.44	1	1.37	0.0431888	*N. megapotamia*
Q37230	Ribulose-1,5-bisphosphate carboxylase/oxygenase large subunit, partial (chloroplast)	48.75	7.05	31.59	1	0.54	0.00334969	*L. humilis*
G0WY03	Ribulose-1,5-bisphosphate carboxylase/oxygenase large subunit, partial (chloroplast)	25.84	6.35	59.74	1	0.81	0.00151791	*B. remotiflorus*
I4EBI1	Ribulose-1,5-bisphosphate carboxylase/oxygenase large subunit, partial (chloroplast)	49.42	6.92	35.79	1	0.06	0.000212022	*C. caricoides*
Q9SAW6	Ribulose-1,5-bisphosphate carboxylase/oxygenase small subunit	18.52	8.56	53.33	1	2.66	0.00865588	*T. aestivum*
W5BMD1	Ribulose bisphosphate carboxylase small chain PW9, chloroplastic	21.23	9.11	67.88	2	1.42	0.00967385	*A. tauschii*
I1I3X4	Ribulose bisphosphate carboxylase small chain PW9, chloroplastic-like	19.44	8.73	27.43	1	0.51	0.00947877	*B. distachyon*
I1IIT7	Ribulose bisphosphate carboxylase small chain PWS4.3, chloroplastic-like isoform 1	19.56	8.73	33.71	1	0.16	0.00314987	*B. distachyon*
K3ZLI3	Ribulose bisphosphate carboxylase/oxygenase activase, chloroplastic-like	51.62	7.74	17.2	1	2.76	0.0258772	*S. italica*
M8CA60	Chlorophyll a-b binding protein 1B, chloroplastic	26.92	5.25	43.14	1	0.80	0.0284038	*A. tauschii*
M0X0F4	Chlorophyll a-b binding protein, chloroplastic	28.26	5.43	28.2	1	0.82	0.0451626	*T. urartu*
M0ZEV8	Chlorophyll a-b binding protein, chloroplastic	24.08	6.33	33.33	1	0.81	0.0343821	*A. tauschii*
W5AG21	Putative chlorophyll a-b binding protein 1C, chloroplastic	7.01	5.91	14.06	1	1.30	0.0156988	*A. tauschii*
M7Z312	Chlorophyll a-b binding protein, chloroplastic	28.28	5.64	17.29	1	0.77	0.00841937	*T. urartu*

Spot no.[a]	Proteins	MV[b]	p*I*[c]	Coverage (%)	Nup.[d]	Ratio[e]	P-value	Plant species
F2CZH5	Cytochrome b6-f complex iron-sulfur subunit	23.73	8.18	43.24	3	1.22	0.0216091	*H.vulgare*
R7W2G6	Cytochrome b6-f complex iron-sulfur subunit, chloroplastic	27.82	6.46	10.57	1	1.22	0.0113752	*A. tauschii*
I1GTJ6	Cytochrome b6-f complex iron-sulfur subunit, chloroplastic-like	23.69	8.25	26.24	1	0.34	0.000989766	*B. distachyon*
A0A0F6NH93	Cytochrome f	35.44	8.91	35.63	1	0.46	0.000176532	*P. virgatum*
M8BGU7	Ferredoxin, chloroplastic	15.04	4.49	31.21	1	1.21	0.0182006	*A. tauschii*
J9QCY8	Ferredoxin-NADP reductase	40.18	7.65	22.87	1	0.56	*0.0103735*	*S. cultivar*
D8L9G3	Sedoheptulose-1,7-bisphosphatase, chloroplast precursor, expressed	42.07	6.57	41.22	1	1.33	0.0206151	*T. aestivum*
P46285	Sedoheptulose-1,7-bisphosphatase, chloroplastic	42.03	6.43	41.22	1	1.63	0.00417048	*T. aestivum*
R7W0K0	Calvin cycle protein CP12	6.89	4.70	31.25	1	1.24	0.00177917	*A. tauschii*
F2DZH9	Carbonic anhydrase	24.64	7.81	12.11	1	0.47	0.000375071	*H. vulgare*
B4F9E2	TPA: Carbonic anhydrase	44.33	8.05	8.42	1	0.77	0.00576693	*Z. mays*
W5FQX4	Chlorophyllase-2, chloroplastic	19.01	6.19	23.43	1	0.80	0.0342267	*T. urartu*
M8BTT2	Protein MRP-like protein	48.17	4.97	21.52	1	0.79	0.0430484	*A. tauschii*
K3XMA8	Protein fluorescent in blue light, chloroplastic-like	20.86	9.01	8.47	1	0.72	0.0452803	*S. Italica*
W5DMP7	Magnesium-protoporphyrin IX methyltransferase	20.25	8.28	21.69	1	0.59	0.0239693	*H. vulgare*
F2CQ53	Proton gradient regulation 7 isoform 1	33.85	5.33	6.77	2	0.83	0.0497281	*H. vulgare*
A3BKU8	Oxygen evolving complex of photosystem II subunit	22.58	9.80	13.15	1	0.45	0.000832852	*O. sativa*
O22575	Glycine decarboxylase P subunit	110.95	6.80	40.64	1	1.41	0.0169824	*x Tritordeum sp.*
**Lipid metabolism**								

Spot no.[a]	Proteins	MV[b]	p*I*[c]	Coverage (%)	Nup.[d]	Ratio[e]	P-value	Plant species
W5BK24	Glycerol-3-phosphate dehydrogenase SDP6, mitochondrial	57.83	7.96	4.21	2	0.79	0.00242913	*T. urartu*
M8B1V8	Phospholipase A1, chloroplastic-like	52.04	6.80	30.38	4	0.83	0.0320995	*A. tauschii*
W5I7C8	Putative glycerophosphoryl diester phosphodiesterase 1	79.71	6.20	5.32	1	1.27	0.0208115	*A. tauschii*
F2DDR0	Acyl activating enzyme	55.82	6.48	9.93	1	1.30	0.0293746	*H. vulgare*
W5GXL1	B-ketoacyl reductase	28.79	9.47	15.15	3	0.81	0.0257862	*H. vulgare*
**RNA metabolism**								
I1HNH2	DEAD-box ATP-dependent RNA helicase 56-like	46.20	6.33	16.83	1	0.50	0.00276501	*B. distachyon*
N1R2R0	33 kDa ribonucleoprotein, chloroplastic	38.54	5.19	16.43	1	1.32	0.0201259	*A. tauschii*
T1N8D9	Ribonucleoprotein chloroplastic-like	28.84	5.36	24.44	4	0.74	0.0229567	*H. vulgare*
J3LP70	Ribonucleoprotein chloroplastic-like	27.93	4.56	14.34	1	0.66	0.000371694	*O. sativa*
M0YI85	Splicing factor	13.54	8.27	5.79	1	0.82	0.0219797	*T. urartu*
M0V1K8	SWIB/MDM2 domain containing expressed	6.59	10.10	18.03	1	0.70	0.00617218	*T. urartu*
**Energy production**								
A9L9Z4	ATP synthase beta subunit	52.96	5.27	58.78	1	1.21	0.000715634	*V. microstachys*
A1E9I7	ATP synthase CF0 B subunit	20.96	9.61	25.14	1	0.72	0.00718473	*T. aestivum*
A0A0F6NQY1	ATP synthase CF1 beta subunit	53.92	5.50	53.41	1	1.46	0.0269899	*S. bicolor*
A0A0E0EPX3	ATP synthase subunit a (chloroplast)	60.31	9.55	14.2	1	1.32	0.048842	*O. rufipogon*
**Cell wall metabolism**								
M8BBJ1	Fasciclin-like arabinogalactan protein 11-like	25.05	9.11	4.49	1	0.71	0.0199912	*A. tauschii*
A0A0A9EJ37	Fasciclin-like arabinogalactan protein 7-like	27.15	7.36	4.23	1	0.82	0.0223486	*S. italica*

Spot no.[a]	Proteins	MV[b]	p*I*[c]	Coverage (%)	Nup.[d]	Ratio[e]	P-value	Plant species
**Lipid metabolism**								
W5FGQ7	Non-specific lipid transfer protein 5	7.54	9.23	53.85	1	0.29	0.00218431	*T. aestivum*
M7YJJ9	Non-specific lipid-transfer protein	11.65	9.04	12.71	1	0.50	0.0322547	*T. urartu*
M8BZN6	Non-specific lipid-transfer protein 2G	9.17	9.26	12.09	1	0.77	0.0162145	*A. tauschii*
W5A6H1	Non-specific lipid-transfer protein 2G	9.15	9.06	27.47	2	0.77	0.00756376	*A. tauschii*
M7ZNW1	Non-specific lipid-transfer protein 2G	9.21	9.07	13.19	1	0.59	0.0062957	*T. urartu*
M8D225	Non-specific lipid-transfer protein 4.3	11.15	9.06	36.52	1	0.53	0.00390157	*A. tauschii*
L7Q3U1	Lipid transfer precursor protein, partial	11.98	9.55	34.71	1	1.54	0.0135667	*T. aestivum*
M8C8N2	Lipid transfer protein	13.32	5.80	7.32	1	0.82	0.00339303	*A. tauschii*
M0VYA0	Lipid transfer protein 7a2b	12.32	9.23	20.49	1	0.49	0.0251947	*H. vulgare*
Q9ATG4	Lipid transfer protein precursor	11.25	9.20	36.52	1	0.82	0.0341824	*T. aestivum*
W5E5K7	Type 1 non-specific lipid transfer protein precursor	7.78	9.23	52.5	1	0.52	0.00049314	*T. aestivum*
Q5NE33	Type 1 non-specific lipid transfer protein precursor	11.11	8.69	43.48	4	0.48	0.00589452	*T. aestivum*
Q9S876	Basic protein WBP1B	9.27	8.87	12.77	1	0.46	0.0244737	*T. Aestivum*
W4ZXN2	Amino acid selective channel protein	11.33	5.50	34.91	1	1.62	0.0374197	*A. tauschii*
M0W593	S-adenosylmethionine mitochondrial carrier protein	21.52	10.74	3.94	1	0.75	0.0110647	*A. Tauschii*
W5EAS7	37 kDa inner envelope membrane protein, chloroplastic	27.65	7.11	26.34	3	0.79	0.0261657	*Tauschii*
M0W5Q8	Probable membrane-associated 30 kDa chloroplastic-like	31.88	9.17	38.87	3	1.20	0.00865255	*T. urartu*
I1GN92	Adipocyte plasma membrane-associated protein-like	43.91	6.55	15.62	1	0.79	0.0024617	*B. distachyon*

Spot no.[a]	Proteins	MV[b]	p*I*[c]	Coverage (%)	Nup.[d]	Ratio[e]	P-value	Plant species
A0A0D9ZF51	Ras-related protein Rab11C-like	23.87	6.93	21.86	3	1.25	0.0272023	*O. sativa*
**Signal transduction**								
K3Y9M3	14-3-3-like protein GF14-12-like	28.41	4.97	26.8	1	0.73	0.0020588	*S. italica*
W5GYA8	Extracellular calcium sensing receptor	33.32	9.50	22.57	1	0.71	0.0248772	*H. vulgare*
W5DSH1	EF hand family expressed	17.42	5.63	13.64	1	0.69	0.0415876	*O. sativa*
M8CAC5	Inositol monophosphatase	10.92	4.78	22.22	2	1.21	0.0252836	*A. tauschii*
M0W468	Probable signal recognition particle 43 kda protein, chloroplastic	24.51	5.17	12.93	1	0.82	0.00634209	*H. distichum*
W5B8U4	Salicylic acid-binding protein 2	28.47	5.01	12.6	1	1.38	0.0308812	*A. tauschii*
**Other metabolisms**								
M8BDB0	Retinol dehydrogenase 13	89.73	6.64	1.37	1	1.22	0.00106059	*A. tauschii*
W5B8E3	Haloalkane dehalogenase 2-like	43.83	7.39	13.22	1	0.66	0.0106465	*A. tauschii*
W5B053	Insulin degrading enzyme	28.97	7.85	5.22	1	1.20	0.0130097	*H. vulgare*
T1L8G1	3-beta hydroxysteroid dehydrogenase isomerase protein	26.51	5.44	43.85	4	1.21	0.00761572	*H. vulgare*
A0A096UKR2	3-beta hydroxysteroid dehydrogenase isomerase protein	31.79	9.03	35.67	4	0.76	0.00472605	*H. vulgare*
W5A3L6	Maf-like protein DDB_G0281937-like	15.86	7.46	10.49	1	1.23	0.0343258	*H. vulgare*
W5FMZ5	Carotenoid cleavage dioxygenase	61.58	6.27	18.07	2	0.78	0.0248091	*B. sylvaticum*
F2CQN3	Divinyl reductase	42.78	6.79	11.56	1	0.82	0.0396977	*H. vulgare*
W5GZW1	Divinyl reductase	26.93	5.27	20.82	2	0.76	0.0154652	*H. vulgare*
A0A0A9JCN5	Chloroplast stem-loop binding protein of 41 kDa chloroplastic-like	22.70	7.91	16.67	3	1.20	0.00760387	*S. bicolor*

[a]Accession no.: accession number in Uniprot database.

[b]Protein MW: molecular mass of predicted protein.

[c]p*I* (kDa): p*I* of predicted protein.

[d]NuP: number of matched unique peptides identified for each protein.

[e]Ratio: the ratio between intensities of identified protein species in treated vs control plants. The ratios that were statistically significant ($p < 0.05$). Ratio changes in expression level of at least 1.2-fold.

Additional Table S3. Comparison of functional classifications of the differentially accumulated proteins between CSP and CTP identified by 2-DE and iTRAQ

Functional categories	No. of DEPs (2-DE)	No. of DEPs (iTRAQ)
Unknown biological processes	3	22
Protein metabolism	2	51
Stress/defense	1	41
Carbohydrate metabolism	1+2	15
Photosynthesis	8+1	38
Energy production	3	4
Nitrogen metabolism	2	2
RNA metabolism	0	6
Sulfur metabolism	0	2
Lipid metabolism	0	5
Cell wall metabolism	0	2
Membrane and transportation	0	19
Signal transduction	0	6
Other metabolic processes	0	10
Totals	20+3	223

DEPs: differentially accumulated proteins
